# NFAT5 dictates crosstalk between intestinal epithelial regenerative capacity and microbiota in murine colitis models

**DOI:** 10.1172/JCI183093

**Published:** 2025-07-15

**Authors:** Se-Hyeon Park, Dae Hee Cheon, Yu-Mi Kim, Yeji Choi, Yong-Joon Cho, Bong-Ki Hong, Sang-Hyun Cho, Mi-Na Kweon, Hyug Moo Kwon, Eugene B. Chang, Donghyun Kim, Wan-Uk Kim

**Affiliations:** 1 Center for Integrative Rheumatoid Transcriptomics and Dynamics, and; 2 Department of Biomedicine and Health Sciences, The Catholic University of Korea, Seoul, South Korea.; 3 Department of Biomedical Sciences, and; 4 Department of Microbiology and Immunology, Seoul National University (SNU) College of Medicine, Seoul, South Korea.; 5 Department of Molecular Bioscience, and; 6 Multidimensional Genomics Research Center, Kangwon National University, Chuncheon, South Korea.; 7 School of Biological Sciences, SNU, Seoul, South Korea.; 8 Mucosal Immunology Laboratory, Department of Convergence Medicine, University of Ulsan College of Medicine/Asan Medical Center, Seoul, South Korea.; 9 Department of Biological Sciences, Ulsan National Institute of Science and Technology, Ulsan, South Korea.; 10 Department of Medicine, University of Chicago IBD Research Center, Chicago, Illinois, USA.; 11 Institute of Endemic Diseases, SNU Medical Research Center, Seoul, South Korea.; 12 Institute of Cancer Research, SNU, Seoul, South Korea.; 13 Division of Rheumatology, Department of Internal Medicine, the Catholic University of Korea, Seoul, South Korea.

**Keywords:** Gastroenterology, Immunology, Inflammatory bowel disease

## Abstract

Hypertonic and hyperosmolar stimuli frequently pose challenges to the intestinal tract. Therefore, a resilient epithelial barrier is essential for maintaining gut homeostasis in the presence of osmotic perturbations. Nuclear factor of activated T cells 5 (NFAT5), an osmosensitive transcription factor, primarily maintains cellular homeostasis under hypertonic conditions. However, the osmoprotective role of NFAT5 in enterocyte homeostasis is poorly understood. Here, we demonstrate that NFAT5 was critical for the survival and proliferation of intestinal epithelial cells (IECs) and that its deficiency accelerated chemically induced or spontaneous colitis in mice. Mechanistically, NFAT5 promoted the survival of IECs and the renewal of intestinal stem cells, thereby regulating the production of mucus and antimicrobial compounds, including RegIII and lysozyme, which consequently shape the gut microbial composition to prevent colitis. Transcriptome analysis identified HSP70 as a key downstream target of NFAT5 in epithelial regeneration. Loss- and gain-of-function experiments involving HSP70 revealed that NFAT5 mitigated experimental colitis through IEC *Hsp70*, which protected stem cells from inflammation-induced injury and maintained barrier function. In conclusion, our study demonstrates what we believe to be a previously unknown role for NFAT5 in dictating the crosstalk between intestinal stem cells and the microbiota, underscoring the importance of the NFAT5/HSP70 axis in maintaining epithelial regeneration related to gut barrier function, balancing microbial composition, and subsequently preventing colitis progression.

## Abstract 2

NFAT5 regulates epithelial regenerative capacity related to gut homeostasis and microbial composition, preventing colitis progression via HSP70.

## Graphical abstract

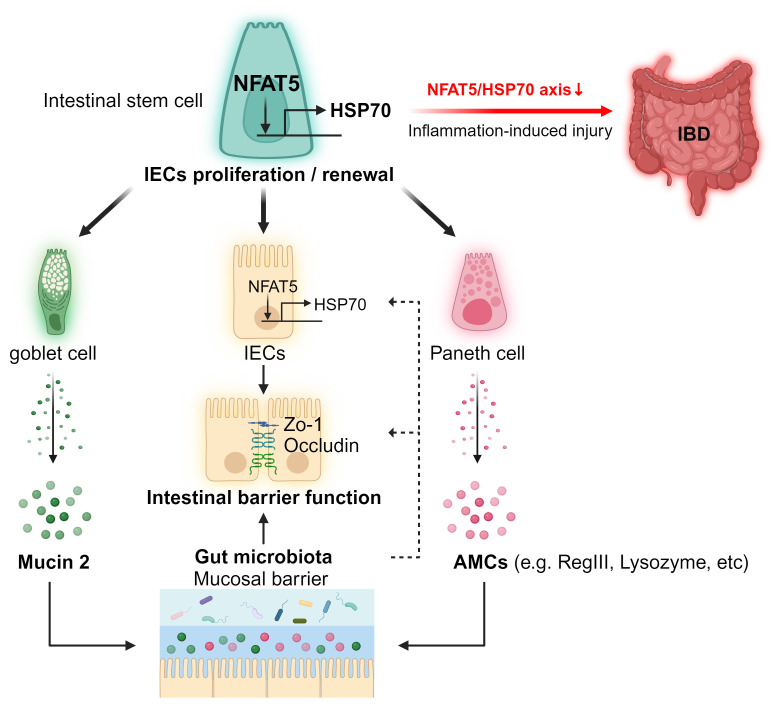

## Introduction

The intestinal epithelium serves as a critical barrier that maintains gut homeostasis by regulating nutrient absorption, immune responses, and microbial interactions (1–3). This single layer of cells undergoes constant renewal, driven by intestinal stem cells (ISCs) located in the crypts, which differentiate into various specialized epithelial cell subtypes, including absorptive enterocytes, mucus-secreting goblet cells, and antimicrobial peptide–producing Paneth cells (4–6). Intestinal epithelial cells are continuously regenerated every 4–5 days through a process of ISC self-renewal, differentiation, and migration, which is essential for maintaining gut barrier integrity (4–7). Disruptions in gut barrier function can lead to increased intestinal permeability, dysbiosis, and susceptibility to inflammatory diseases such as inflammatory bowel disease (IBD), which encompasses Crohn’s disease and ulcerative colitis (1, 2).

IBD is characterized by chronic, relapsing inflammation of the gastrointestinal tract, often linked to a combination of genetic susceptibility, environmental factors, immune dysregulation, and alterations in the gut microbiota (1, 2, 8). The intestinal epithelium actively orchestrates immune responses and maintains symbiotic interactions with the gut microbiota, which comprise bacteria, archaea, protists, fungi, and viruses (4, 9). These commensal microbes influence mucosal immune responses, metabolize dietary components into nutrients and metabolites, and reinforce epithelial barrier integrity (1, 8, 10). Disruptions in this delicate balance can lead to microbial dysbiosis, which exacerbates inflammation and compromises epithelial function, thereby perpetuating IBD pathology (8, 10, 11). Understanding the intricate molecular mechanisms that regulate epithelium integrity and microbial composition is therefore crucial for identifying new therapeutic targets for IBD (2, 4, 11, 12).

Recent advances in gut microbiome research have revealed the complex interplay between host genetics, microbial diversity, and disease susceptibility (1, 3, 4, 8, 10). Studies suggest that alterations in gut microbial composition influence disease progression by modulating immune responses and altering epithelial barrier function (1, 3, 4, 10, 11). In particular, beneficial microbes, such as members of the *Lachnospiraceae* and *Faecalibacterium* genera, play key roles in maintaining intestinal homeostasis by producing short-chain fatty acids (SCFAs) and regulating tight-junction proteins (8, 11, 13). Conversely, an increase in pathogenic bacteria, such as adherent-invasive *Escherichia coli* (AIEC) and certain *Bacteroides* species have been associated with gut barrier dysfunction and excessive inflammatory responses in patients with IBD (1, 8, 11, 14). These findings highlight the importance of microbe-epithelium interactions in gut health and disease.

Nuclear factor of activated T cells 5 (NFAT5), also known as the tonicity-responsive enhancer-binding protein (TonEBP), primarily maintains cellular homeostasis under hypertonic conditions, particularly in the kidneys, by orchestrating the transcriptional activation of osmoprotective genes (15–17). Given that the intestinal lumen is frequently subjected to osmotic fluctuations caused by dietary intake, microbial metabolites, and host-secreted factors, NFAT5 may be critically involved in maintaining epithelial resilience under these conditions (15, 16, 18). Interestingly, isotonic stimuli can also trigger NFAT5 expression and activation (19–22). We and others have reported the isotonic functions of NFAT5 in regulating the production of various proinflammatory cytokines, as well as the migration, survival, and proliferation of macrophages (19–21), which may further link the role of NFAT5 to inflammation-associated diseases such as IBD. Moreover, hyperosmotic stress has been implicated in inflammatory signaling in intestinal epithelial cells (IECs), suggesting that NFAT5 may act as a sensor that integrates osmotic stress with immune regulation in the gut. However, the role of NFAT5 in intestinal stem cell maintenance, as well as its precise contributions to epithelial barrier function and host-microbiota interactions, has not been clearly defined.

We postulate that the intestine adapts to the hypertonic and hyperosmolar microenvironments within the digestive tract by increasing the levels of the osmosensitive transcription factor NFAT5. In this study, we demonstrate that NFAT5 was required for the survival and proliferation of IECs and that its deficiency accelerated experimental colitis in mice. This function of NFAT5 was achieved through the regulation of both the gut microbiota and intestinal regeneration, which are necessary for IEC reconstruction. Moreover, as a key target gene of NFAT5, heat shock protein 70 (*HSP70*) mediates the survival and proliferation of IECs and protects against colitis. In summary, our research provides insights into the alleviation of experimental colitis by NFAT5 via IEC *HSP70*, a process that safeguards stem cells and maintains intestinal barrier integrity against inflammation-induced injury.

## Results

### NFAT5 deficiency reduces IEC survival and proliferation.

**Figure 1:**
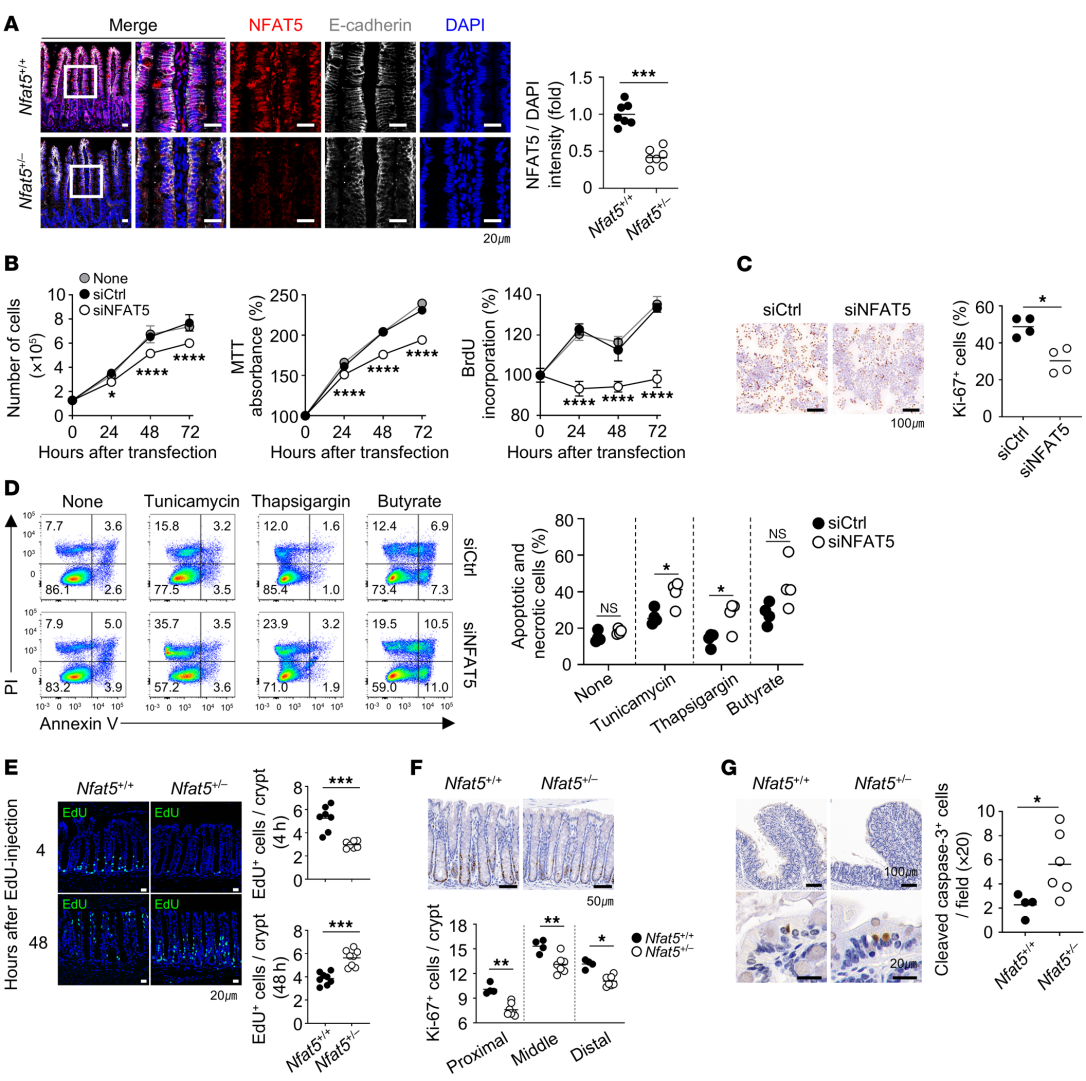
NFAT5 promotes the proliferation and survival of IECs. (**A**) Representative images and a corresponding graph of immunofluorescence staining of ileal tissue from *Nfat5^+/+^* and *Nfat5^+/–^* mice for NFAT5 (red) and E-cadherin (white). Nuclei are counterstained with DAPI (blue). Scale bars: 20 μm. (**B** and **C**) Proliferation of HT-29 cells transfected with siCtrl or siNFAT5 was assessed at the indicated time points using trypan blue exclusion (left), MTT (middle), and BrdU incorporation assays (right) (**B**). Representative images of Ki-67 immunocytochemistry and quantification of Ki-67^+^ cells after 48 hours of transfection with siCtrl or siNFAT5 are shown (**C**). Scale bars: 100 μm. (**D**) Flow cytometric analysis of annexin V and PI staining of HT-29 cells transfected with siCtrl or siNFAT5 for 48 hours, followed by 24 hours of treatment with thapsigargin, tunicamycin, or butyrate. The total frequency of apoptotic and necrotic cells is shown (annexin V^–^/PI^–^, live cells; annexin V^–^/PI^+^, necrotic cells; annexin V^+^/PI^–^, early apoptotic cells; annexin V^+^/PI^+^, late apoptotic cells). (**E** and **F**) Proliferating cells in distal colonic tissues of *Nfat5^+/+^* and *Nfat5^+/–^* mice were assessed by EdU-incorporation assay (**E**) and Ki-67 IHC (**F**). EdU^+^ cells were detected after intraperitoneal injection of 1 mg EdU at the indicated time points. Representative images and quantified data are shown. Scale bars: 20 μm (**E**) and 50 μm (**F**). (**G**) Apoptotic cells were determined by cleaved caspase 3 IHC in proximal colonic tissues of *Nfat5^+/+^* and *Nfat5^+/–^* mice after 3 days of DSS treatment. Representative images and quantification of cleaved caspase 3^+^ cells are shown. Scale bars: 100 μm (top panels) and 20 μm (bottom panels). Each dot represents an individual mouse, and the means are displayed as a line (**A** and **E**–**G**). Data are presented as the mean ± SD (**B**) and as a line indicating the mean (**C**). *P < 0.05, **P < 0.01, ***P < 0.001, and ****P < 0.0001 by Mann-Whitney *U* test (**A**, **C**, and **E**–**G**) and 2-way, repeated-measures ANOVA with Šídák’s multiple-comparison test (between cells transfected with siCtrl and siNFAT5; **B** and **D**). Data shown in **A**–**G** are representative of at least 2 independent experiments.

We first found that NFAT5 protein, which is known to be expressed in various cell types, including those of the kidney, cancer cells, and immune cells (23), was also expressed in the intestinal epithelium of WT mice (Figure 1A). However, its expression was significantly downregulated in the intestinal epithelium of *Nfat5*-deficient (*Nfat5^+/–^*) mice, as shown by a comparative analysis of intestinal tissues from *Nfat5^+/–^* mice and WT littermates via immunofluorescence staining (Figure 1A). Given that complete KO of *Nfat5* is lethal (24), we used haploinsufficient mice, in which a single *Nfat5* allele is deleted. NFAT5 is required for the survival and proliferation of diverse types of cells, including kidney epithelial cells, cancer cells, T cells, and macrophages (15, 17, 19, 25). To determine the involvement of NFAT5 in IEC functions, we downregulated its expression in the human colorectal adenocarcinoma cell lines HT-29 and Caco-2 using siRNAs (Supplemental Figure 1, A and B; supplemental material available online with this article; https://doi.org/10.1172/JCI183093DS1). Under normal culture conditions, the *NFAT5*-knockdown cells had a significantly lower rate of cell proliferation than did the untransfected or scrambled siRNA–transfected cells (Figure 1, B and C, and Supplemental Figure 1, C and D). In the presence of endoplasmic reticulum (ER) stress inducers, such as thapsigargin and tunicamycin, and of apoptotic cell death inducers, such as sodium butyrate, *NFAT5* knockdown rendered the cells significantly more susceptible to cell death (Figure 1D and Supplemental Figure 1E). Moreover, consistent with these results, 5-ethynyl-2′-deoxyuridine (EdU) pulse labeling and immunostaining for Ki-67 and cleaved caspase 3 revealed reduced IEC proliferation and a higher number of apoptotic cells in the colon epithelia of *Nfat5^+/–^* mice compared with their WT littermates (Figure 1, E–G). These findings indicate an intrinsic role of NFAT5 in IEC proliferation and survival.

### Nfat5 knockdown accelerates experimental colitis in mice.

**Figure 2:**
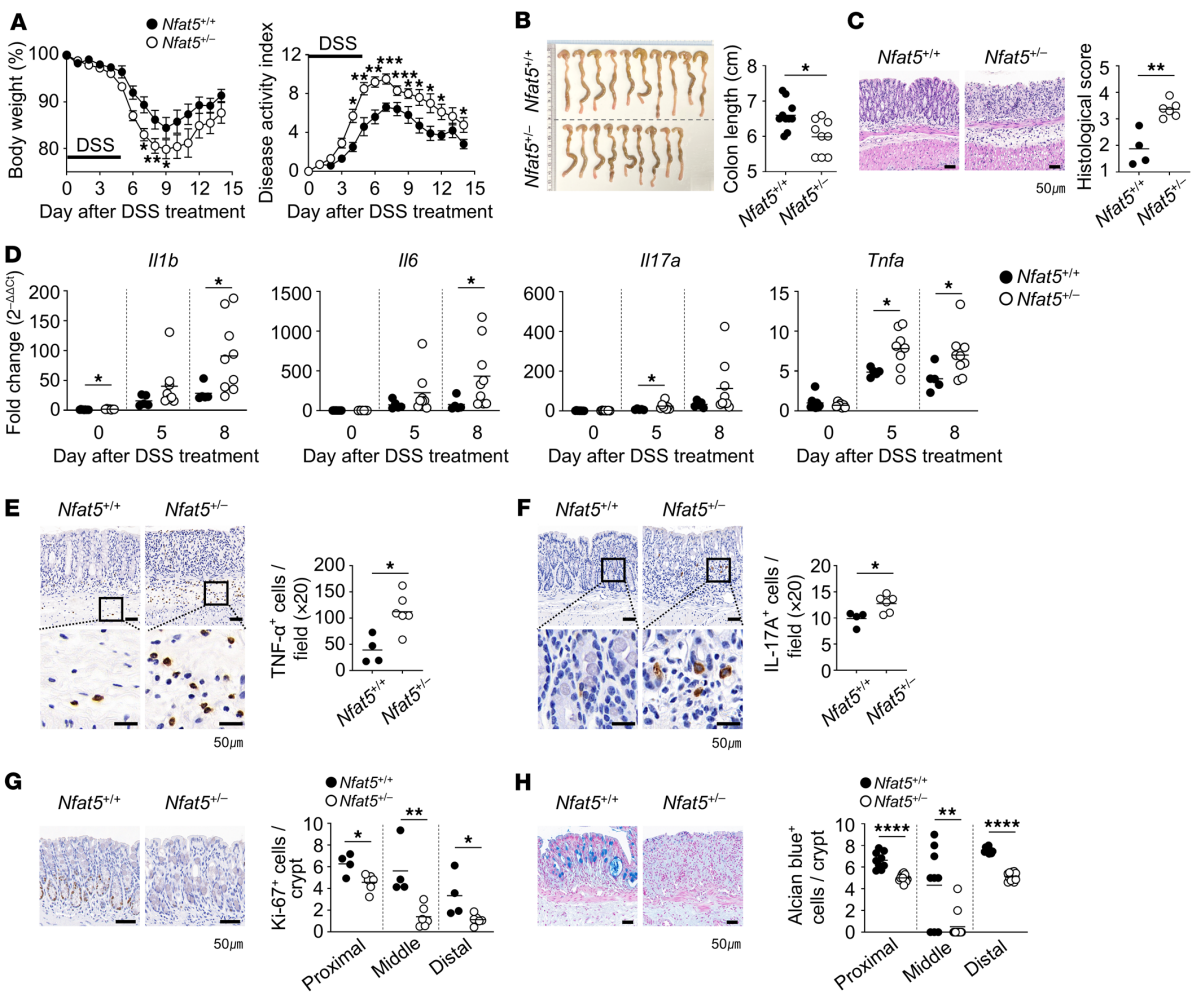
NFAT5 protects mice against DSS-induced colitis. *Nfat5^+/+^* and *Nfat5^+/–^* mice were given ad libitum access to water containing DSS for 5 days, after which fresh water was made available for the remainder of the experimental period. (**A** and **B**) Body weight changes and the DAI were monitored for 14 days after the initial DSS treatment (**A**), and on day 14, macroscopic images and colon lengths on day 14 were analyzed (*n* = 10 per group) (**B**). One *Nfat5^+/–^* mouse died on day 13. DAI scores were determined in accordance with the criteria outlined in Supplemental Table 1. (**C**) H&E staining of the distal colonic tissues collected on day 5 after DSS treatment. Representative H&E images are shown, and the histological score was calculated, as shown in Supplemental Table 3. Scale bars: 50 μm. (**D**) Relative mRNA expression of *Il1b*, *Il6*, *Il17a*, and *Tnfa* in whole colonic tissues at the indicated time points were determined by qRT-PCR. *Gapdh* mRNA levels were used as an internal control. (**E**–**H**) IHC staining was performed on colonic tissues harvested on day 5 to evaluate the presence of TNF-α^+^ (**E**), IL-17^+^ (**F**), Ki-67^+^ (**G**), and Alcian blue^+^ (mucin-producing) (**H**) cells. Representative images of the distal colon and the corresponding graphs are shown (scale bars: 50 μm). TNF-α^+^ and IL-17^+^ cells were counted in at least 5 fields of the distal colon per mouse. Ki-67^+^ cells and Alcian blue^+^ cells were counted in at least 20 crypts per mouse. Data are presented as the mean ± SEM. Each dot represents an individual mouse, and the means are displayed as lines. Data in **A**–**H** are representative of at least 2 independent experiments. *P < 0.05, **P < 0.01, ***P < 0.001, and ****P < 0.0001, by 2-way, repeated-measures ANOVA with Šídák’s multiple-comparison test (**A**) and Mann-Whitney *U* test (**B**–**H**).

On the basis of the in vitro results, we investigated the role of NFAT5 in the progression of intestinal mucosal injury. To this end, we used dextran sulfate sodium (DSS) to induce colitis in *Nfat5*-haploinsufficient (*Nfat5^+/–^*) mice and their WT (*Nfat5^+/+^*) littermates. The *Nfat5^+/–^* mice showed more severe body weight loss, had a higher disease activity index (DAI), and had shorter colons than did the control littermates (Figure 2, A and B). Consistent with these results, we observed a more severe pathology in the distal colons of *Nfat5*-deficient mice (Figure 2C). The colonic tissues of these mice also showed significantly higher mRNA and protein expression of proinflammatory cytokines, such as *Il1b*, *Il6*, *Il17a*, and *Tnfa*, than did the tissues of their WT counterparts (Figure 2, D–F). *Nfat5* knockdown consistently led to a marked loss of proliferating epithelial cells and mucus-producing cells in mice with DSS-induced colitis (Figure 2, G and H), as determined by Ki-67 and Alcian blue staining, respectively. Together, these results suggest that NFAT5 contributed to protection against the progression of experimental colitis.

### Both epithelial NFAT5 levels and gut microbiota influence the progression of DSS-induced colitis.

Crosstalk between the gut microbiota and IECs is essential for maintaining gut homeostasis (2). To determine the role of the gut microbiota in the deterioration of colitis in *Nfat5*-deficient mice, we first compared the severity of DSS-induced colitis by evaluating weight loss and the DAI score for mice that were either housed separately or cohoused for more than 6 weeks. As expected, we noted more severe colitis symptoms in *Nfat5*-deficient mice than in their control littermates when they were housed separately (Supplemental Figure 2, A, B, and E). However, when cohoused, the 2 groups showed no significant differences (Supplemental Figure 2, C, D, and F). Similarly, mortality was higher for *Nfat5^+/–^* mice than for WT mice when housed separately, but this was almost completely reversed by cohousing the 2 groups (Supplemental Figure 2G). Consistent with this, the DSS-induced increase in gut permeability, as determined by a FITC-dextran permeability assay in *Nfat5^+/–^* mice, was completely restored through cohousing (Supplemental Figure 2, H and I). Collectively, the comparison of data between separately housed and cohoused mice suggests that gut microbiota is necessary for protection against DSS-induced colitis aggravated by *Nfat5* deficiency.

Next, to clarify the role of NFAT5 in IECs during DSS-induced colitis, we generated conditional-KO mice lacking *Nfat5* specifically in IECs using the Cre/loxP system. As expected, we confirmed that in the *Vil-Cre* *Nfat5^fl/fl^* (*Nfat5^IEC-KO^*) mice, *Nfat5* mRNA levels were reduced only in IECs and not in mesenteric lymph nodes, as compared with *Nfat5^fl/fl^* mice (Cre recombinase–negative mice) (Supplemental Figure 3A). Additionally, NFAT5 protein expression was also markedly diminished in the intestinal epithelium regardless of IEC subtype, thus confirming the depletion of *Nfat5* specifically in IECs (Supplemental Figure 3, B–E). Similar to *Nfat5^+/–^* mice, we found that *Nfat5^IEC-KO^* mice had delayed replenishment of IECs, as evidenced by decreased EdU positivity in crypts at 4 hours and belatedly increased EdU positivity at 48 hours following intraperitoneal EdU injection (Supplemental Figure 3F). By contrast, WT mice had a higher number of EdU-stained cells in the crypts early on, with rapid migration toward the villi over time. These mice also exhibited reduced proliferation, as shown by a decrease in Ki-67^+^ IECs, and increased apoptosis, as indicated by elevated cleaved caspase 3^+^ IECs, confirming in vivo that epithelial *Nfat5* deficiency repressed the proliferation and survival of intestinal epithelial cells (Supplemental Figure 3, G and H).

**Figure 3:**
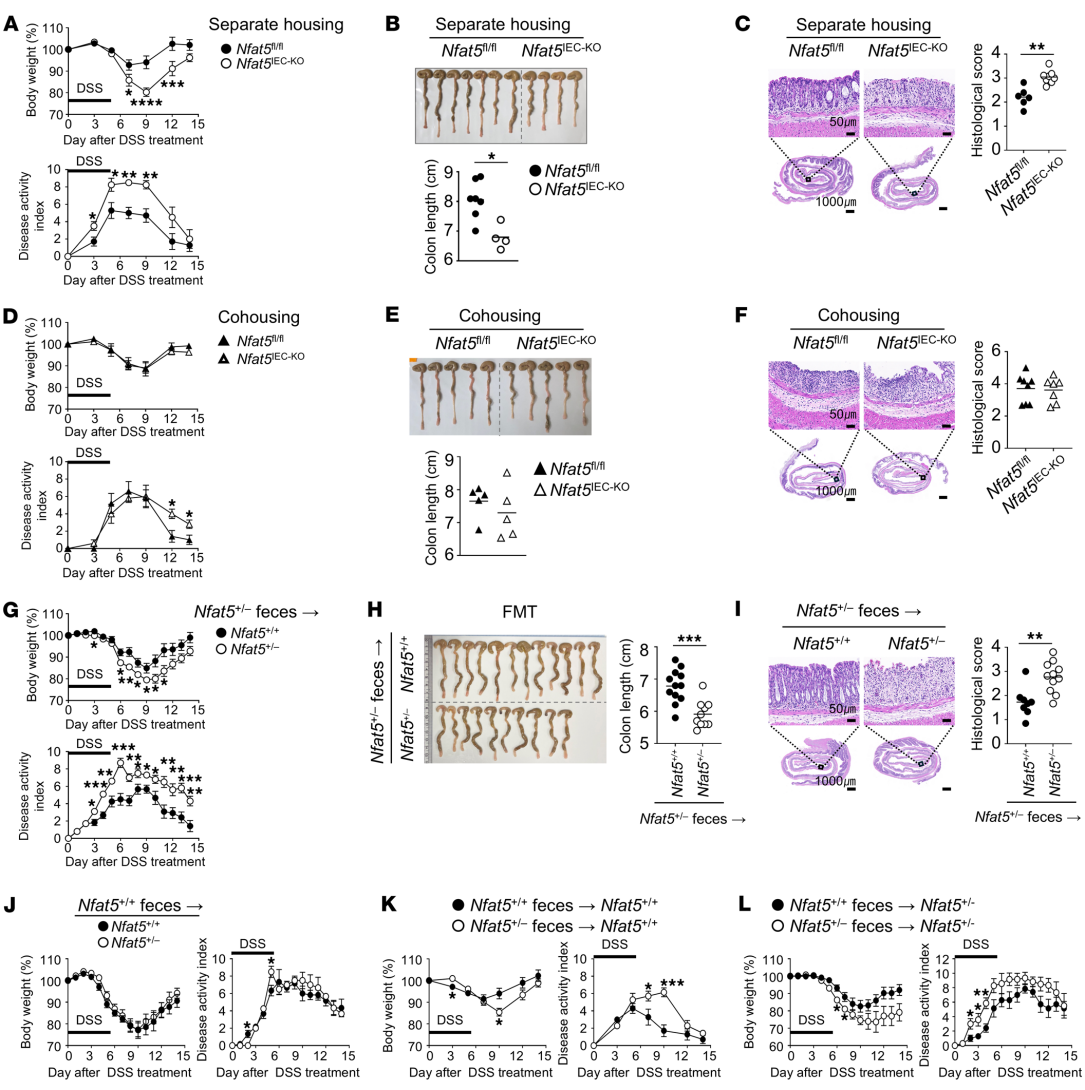
Fecal microbiota are indispensable for driving the progression of colitis associated with NFAT5 deficiency. Mice were given ad libitum access to water supplemented with DSS for 5 days, followed by fresh drinking water for the duration of the experimental period. (**A**–**F**) Body weight changes and DAI scores of separately housed (**A**) and cohoused (**D**) *Nfat5^fl/fl^* and *Nfat5^IEC-KO^* mice. Day-14 macroscopic images of separately housed (**B**) and cohoused (**E**) *Nfat5^fl/fl^* and *Nfat5^IEC-KO^* mice, along with analyses of colon lengths. On day 5, H&E staining was performed on colonic tissues from separately housed (**C**) and cohoused (**F**) *Nfat5^fl/fl^* and *Nfat5^IEC-KO^* mice, and corresponding histological scores were assessed, as detailed in Supplemental Table 3. (**G**–**L**) Recipient mice were pretreated ad libitum with an antibiotic cocktail for 3 weeks and then transplanted 3 times, every other day, with feces obtained from donor mice. DSS was then provided ad libitum for 5 days, followed by fresh drinking water for 9 days. Body weight changes and the DAI were assessed for *Nfat5^+/+^* and *Nfat5^+/–^* recipient mice (**G**), which were transplanted with feces derived from *Nfat5^+/–^* donor mice. (**H**) Macroscopic images and colon lengths were analyzed on day 14. (**I**) H&E-staining of distal colonic tissues collected on day 5, along with corresponding histological scores were evaluated. (**J**–**L**) Body weight changes and the DAI in different combinations (shown in indices) of donor mouse feces and FMT recipient mice were evaluated for the indicated durations. Data are presented as the mean ± SEM (**A**, **D**, **G**, and **J**–**L**). Data shown in **A**–**L** are representative of at least 2 independent experiments. Each dot represents an individual mouse, with mean values indicated by lines (**B**, **C**, **E**, **F**, **H**, and **I**). Scale bars: 1,000 μm and 50 μm (enlarged insets) (**C** and **I**). *P < 0.05, **P < 0.01, ***P < 0.001, and ****P < 0.000, by 2-way, repeated-measures ANOVA with Šídák’s multiple-comparison test (**A**, **D**, **G**, and **J**–**L**) and Mann-Whitney *U* test (**B**, **C**, **E**, **F**, **H**, and **I**).

Moreover, *Nfat5^IEC-KO^* mice exhibited more severe DSS-induced colitis than did *Nfat5^fl/fl^* (Cre recombinase–negative) mice, as determined by body weight loss, DAI score, macroscopic colon shortening, and microscopic pathology results (Figure 3, A–C), demonstrating the critical role of epithelial NFAT5 in aggravating DSS-induced colitis. In contrast, consistent with the findings in *Nfat5^+/–^* and WT mice, there were no differences in the symptoms of DSS-induced colitis between the cohoused *Nfat5^IEC-KO^* and *Nfat5^fl/fl^* mice, suggesting the essential role of gut microbiota in protecting against *Nfat5* deficiency–accelerated colitis (Figure 3, D–F). To further investigate this, we conducted fecal microbiota transplantation (FMT) experiments in *Nfat5^+/–^* and *Nfat5^+/+^* mice. After separate housing in addition to distinct microbiome differences associated with NFAT5 expression, our findings from separate housing and cohousing experiments using FMT from *Nfat5^+/–^* mice into *Nfat5^+/–^* mice resulted in higher colitis severity than that observed in WT (*Nfat5^+/+^*) mice (Figure 3, G–I). This supports notion that the NFAT5 expression level in the host (recipients), which originates from genetic differences, determines the development of DSS-induced colitis. Meanwhile, the increased colitis severity observed in separately housed *Nfat5^+/–^* mice (Supplemental Figure 2A) was almost completely reversed by FMT using feces from WT donors (Figure 3J), suggesting that normal gut microbiota —presumably containing beneficial microbial components — can mitigate DSS-induced colitis in *Nfat5*-deficient recipients by counteracting pathobionts present in *Nfat5^+/–^* mice. In support of this notion, when transplanted into either WT or *Nfat5^+/–^* recipients, feces of *Nfat5^+/–^* mice were more effective at inducing DSS colitis than were feces from WT mice (Figure 3, K and L). Together, these observations suggest that both the gut microbiota and the integrity of IECs, compromised by *Nfat5* deficiency, contribute to the progression of DSS-induced colitis.

### NFAT5 promotes intestinal barrier function through the regulation of gut microbiota.

**Figure 4:**
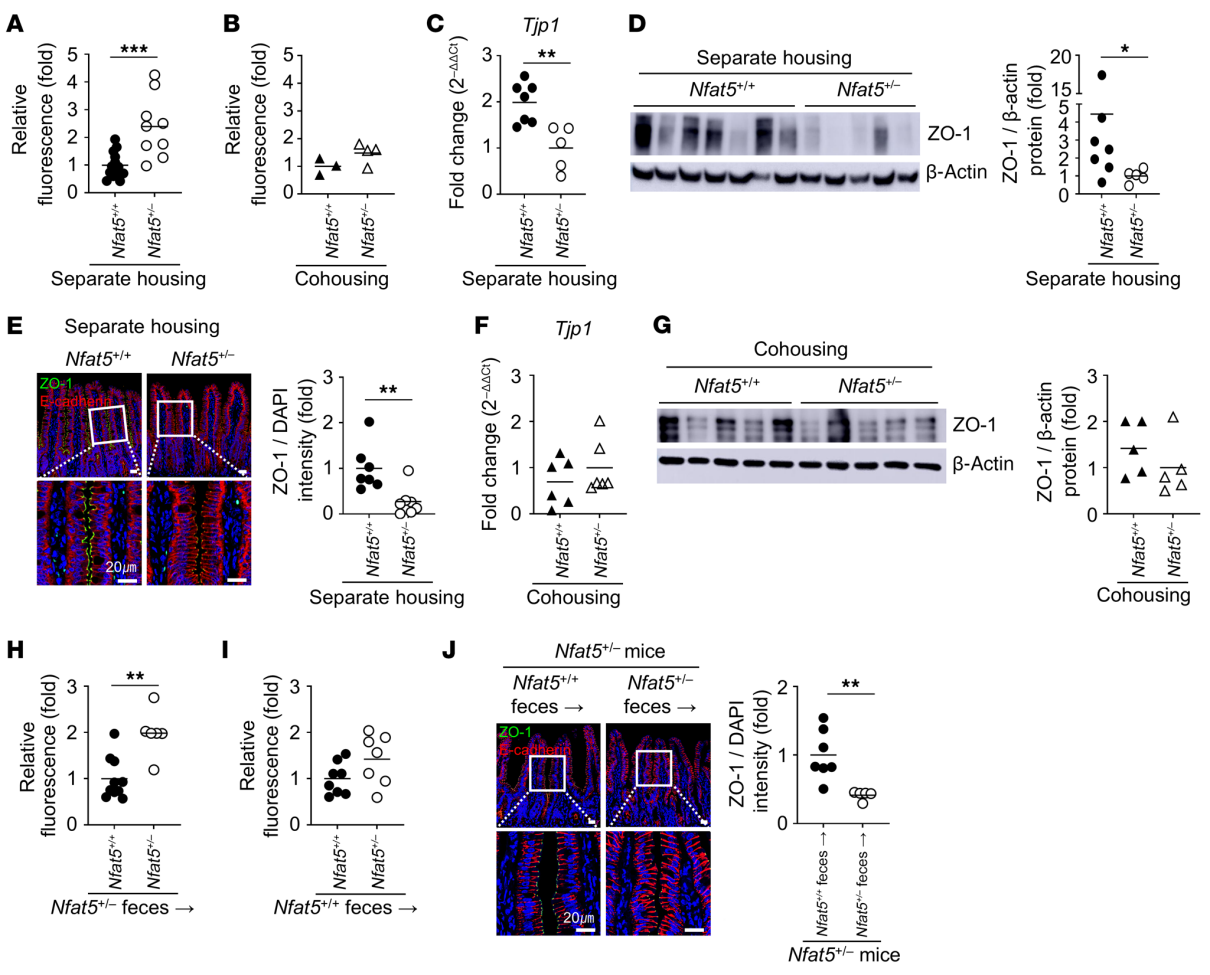
Gut permeability and the expression of tight-junction molecules are modulated by *Nfat5* deficiency and exposure to feces from *Nfat5^+/–^* mice. (**A** and **B**) Results of a gut permeability assay in *Nfat5^+/+^* and *Nfat5^+/–^* mice housed separately (**A**) or cohoused (**B**). Mice were fasted for 12 hours, after which FITC-dextran (average molecular weight ≈4 kDa) was administered to the mice via oral gavage. Four hours later, fluorescence signals were measured in sera of the mice. (**C** and **D**) Relative expression levels of *Tjp1* (ZO-1) mRNA and protein in small IECs from separately housed *Nfat5^+/+^* and *Nfat5^+/–^* mice were determined by qRT-PCR and immunoblotting, respectively. *Gapdh* (**C**) and β-actin (**D**) were used as internal controls, respectively. (**E**) ZO-1 expression in the ileal tissues of separately housed *Nfat5^+/+^* and *Nfat5^+/–^* mice was assessed by immunostaining with anti-ZO-1 (green) and anti–E-cadherin (red) antibodies. Nuclei were counterstained with DAPI (blue). Representative merged images and corresponding graphs are shown. Scale bars: 20 μm. (**F** and **G**) Relative *Tjp1* (ZO-1) mRNA and protein expression levels in small IECs of cohoused *Nfat5^+/+^* and *Nfat5^+/–^* mice by qRT-PCR and immunoblotting, respectively. *Gapdh* (**F**) and β-actin (**G**) were used as internal controls for normalization, respectively. (**H**–**J**) Recipient mice were pretreated 5 times, every other day with an antibiotic cocktail for 3 weeks and transplanted with feces obtained from donor mice (shown in indices). A gut permeability assay was performed on *Nfat5^+/+^* and *Nfat5^+/–^* recipient mice transplanted with fecal microbiota derived from *Nfat5^+/–^* (**H**) and *Nfat5^+/+^* donors (**I**). Fluorescence signals were measured in their sera after oral gavage of FITC-dextran. The expression of ZO-1 in the ileal tissues of *Nfat5^+/–^* mice, following fecal transplantation from either *Nfat5^+/+^* or *Nfat5^+/–^* donors, was assessed by immunostaining using anti–ZO-1 (green) and anti–E-cadherin (red) antibodies (**J**). Scale bars: 20 μm. Nuclei were counterstained with DAPI (blue). Representative merged images and corresponding graphs are shown. Each dot represents an individual mouse, and the means are displayed as lines (**A**–**J**). *P < 0.05, **P < 0.01, and ***P < 0.001, by Mann-Whitney *U* test. Data shown in **A**–**J** are representative of 2 independent experiments.

Next, we examined the effect of *Nfat5* deficiency and gut microbes on intestinal barrier function to understand the mechanisms underlying *Nfat5*- and microbiota-mediated protection against colitis. Strikingly, we observed that the gut of *Nfat5^+/–^* mice became more permeable than that of WT mice, even in the absence of colitis, solely by raising the mice in separate cages according to their genotype (Figure 4A), indicating that a genetic defect in NFAT5 could impair the normal function of the physiological gut barrier. Interestingly, such an increase in gut permeability was substantially attenuated by cohousing *Nfat5^+/–^* and WT mice (Figure 4B), suggesting the additional involvement of gut microbiota in maintaining gut barrier function. In line with this, the mRNA and protein expression levels of zonula occludens 1 (ZO-1) (*Tjp1*) and occludin (*Ocln*), well-known tight-junction molecules (12), were also remarkably lower in *Nfat5*-lacking IECs of separately housed mice (Figure 4, C–E, and Supplemental Figure 4, A and B), a difference that was almost completely abolished by cohousing (Figure 4, F and G, and Supplemental Figure 4C). Moreover, when feces from *Nfat5^+/–^* mice were transplanted, the *Nfat5*-deficient gut still exhibited higher permeability than did the *Nfat5*-sufficient gut (Figure 4H). However, when feces from WT donors were transplanted, the *Nfat5^+/–^* recipient mice showed an improvement in gut permeability similar to that of WT recipient mice (Figure 4I). In parallel, FMT using feces from *Nfat5^+/+^* mice significantly restored the expression of tight-junction proteins, such as ZO-1 and occludin, in the intestines of *Nfat5^+/–^* mice compared with FMT using feces from *Nfat5^+/–^* mice (Figure 4J and Supplemental Figure 4D). These data indicate that NFAT5 plays a pivotal role in preserving intestinal homeostasis and that microbiota alterations resulting from *Nfat5* deficiency have a direct influence on gut barrier function.

**Figure 5:**
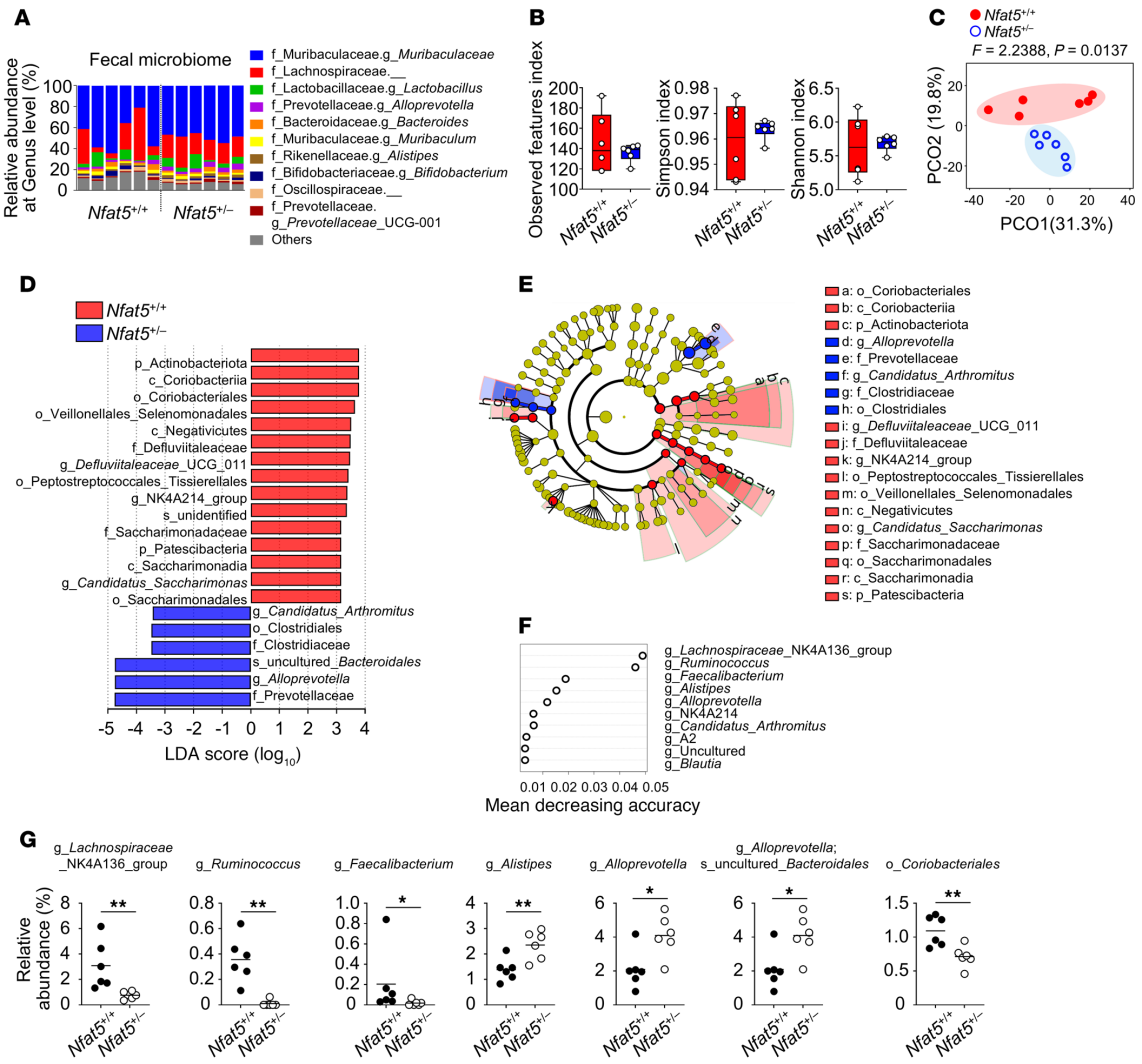
NFAT5 deficiency alters the gut microbiome. (**A**–**F**) The fecal microbiome of separately housed *Nfat5^+/+^* or *Nfat5^+/–^* mice (*n* = 6 per group) was analyzed by 16S rRNA amplicon sequencing. Relative abundance of fecal bacteria at the genus level (**A**), observed features, Simpson’s and Shannon’s indices for α-diversity (**B**), PCOA plot at amplicon sequence variant level for β-diversity (**C**), LEfSe analysis (**D**) (LDA score >3), random forest analysis at the genus level (**E**), and the relative abundance of each taxon highly suggested from the results of **A** and **D** (**F** and **G**). Pseudo-*F* and *P* values in **C** were analyzed by PERMANOVA. Data in **B** are presented as box-and-whisker plots (minimum to maximum, with a line at the median). Each dot in **G** represents an individual mouse, with mean values indicated by lines. *P < 0.05 and **P < 0.01, by Mann-Whitney *U* test.

To further support this assumption, we performed 16S ribosomal RNA amplicon sequencing to assess the microbial composition in feces and cecum contents obtained from *Nfat5^+/–^* and WT mice that were raised separately. The MiSeq system provided 310,555 and 287,009 qualified sequences (median: 23,132.5 and 25,414 reads per sample; range: 22,129-32,317 and 16,510-35,926) of 16S rRNA amplicons from fecal and cecal samples, respectively. The microbial taxa at the genus level were dominated by *Muribaculaceae*, an undefined taxon (belonging to the family *Lachnospiraceae*), and by *Lactobacillus* in the feces and an unidentified taxon (belonging to the family *Lachnospiraceae*), *Muribaculaceae*, and by the *Lachnospiraceae* NK4A136 group in the cecal contents (Figure 5A and Supplemental Figure 5A). There were no significant differences in species numbers or evenness in the feces between *Nfat5^+/–^* and WT mice, as determined by the observed features, Simpson, and Shannon indices (Figure 5B). However, according to the observed features index, the cecal contents of the *Nfat5^+/–^* mice had a higher total number of species than did those of WT mice (Supplemental Figure 5B). Of note, through principal coordinate analysis (PCOA), we observed clear and distinct clusters between *Nfat5^+/–^* and WT mice in both feces and cecal contents (Figure 5C and Supplemental Figure 5C), indicating that *Nfat5* deficiency had a notable effect on the microbial composition of both feces and cecal contents. Given that alterations in the gut microbiota of *Nfat5*-deficient mice were detected in the small intestine prior to the arrival of luminal contents in the colon, these findings support the notion that dysbiosis may originate in the small intestine.

Given the aforementioned findings, we sought to identify the specific microbial components that differed between the 2 genotypes. We identified 21 microbial taxa and the top 10 genera using linear discriminant analysis (LDA) effect size (LEfSe) and random forest analysis, respectively (Figure 5, D and E). At the genus level, the *Lachnospiraceae* NK4A136 group as well as *Ruminococcus* and *Faecalibacterium* were enriched in the feces of WT mice, whereas *Alistipes* and *Alloprevotella* were dominant in the feces of *Nfat5*-deficient mice (Figure 5F and data not shown). Meanwhile, the order of *Coriobacteriales* was abundant in the feces of WT mice, whereas the species of uncultured *Bacteroidales* belonging to *Alloprevotella* was abundant in the feces of *Nfat5^+/–^* mice (Figure 5, F and G). Similarly, we identified significantly different taxa in the cecal contents of *Nfat5^+/–^* mice and WT mice (Supplemental Figure 5, D and E).

In summary, these findings demonstrate that *Nfat5* deficiency altered the gut microbiota, presumably contributing to intestinal barrier dysfunction and increased susceptibility to DSS-induced colitis. Such microbial shifts appeared to stem from alterations in the small intestine.

### Nfat5 deficiency impairs IEC regeneration and reduces goblet and Paneth cells, leading to gut barrier dysfunction.

**Figure 6:**
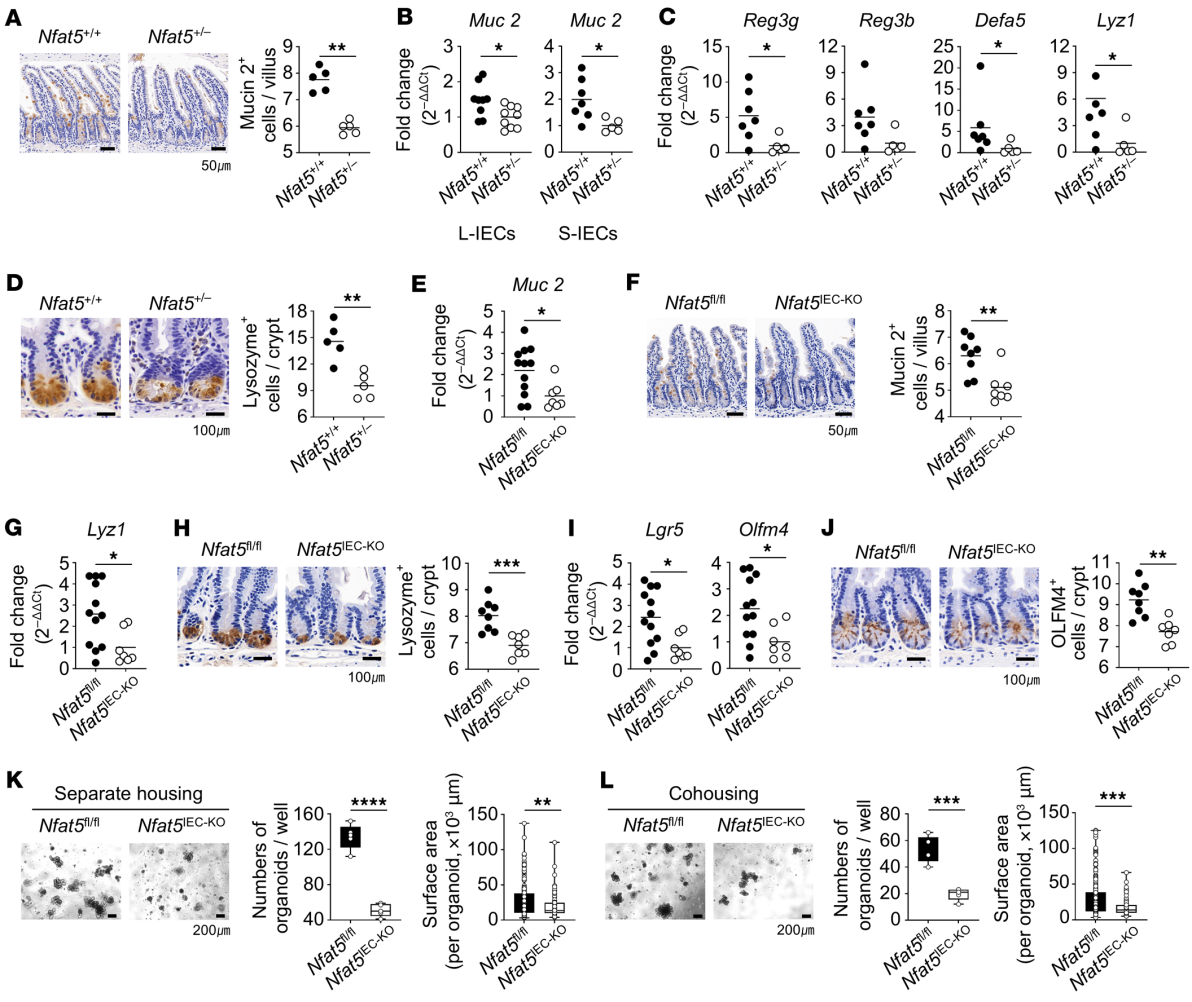
NFAT5 facilitates mucin and antimicrobial compound production in goblet and Paneth cells by regulating epithelial regenerative capacity. (**A**) Mucin 2–expressing cells in the ileal tissues of separately housed *Nfat5^+/+^* and *Nfat5^+/–^* mice were assessed by IHC staining. Representative images and the corresponding graph are presented. Scale bars: 50 μm. (**B** and **C**) *Muc 2* mRNA expression was analyzed using qPCR in large IECs (L-IECs) and small IECs (S-IECs) from separately housed *Nfat5^+/+^* and *Nfat5^+/–^* mice (**B**). Additionally, mRNA expression levels of *Reg3g*, *Reg3b*, *Defa5*, and *Lyz1* were assessed in S-IECs from mice of both groups (**C**), with *Gapdh* mRNA serving as the internal control. (**D**) Lysozyme-expressing cells in the ileal tissues of separately housed *Nfat5^+/+^* and *Nfat5^+/–^* mice were assessed. Representative images and their corresponding graph are presented. Scale bars: 100 μm. (**E**–**J**) Expression levels of mucin 2, lysozyme, LGR5, and OLFM were determined by qRT-PCR and immunostaining. *Muc2* (**E**), *Lyz1* (**G**), *Lgr5*, and *Olfm4* (**I**) mRNA expression levels were measured in S-IECs of separately housed *Nfat5*^fl/fl^ and *Nfat5^IEC-KO^* mice by qRT-PCR; *Gapdh* was used as an internal control for normalization. Mucin 2^+^ (**F**), lysozyme^+^ (**H**), and OLFM4^+^ (**J**) cells were analyzed in the ileal tissues of separately housed *Nfat5^fl/fl^* and *Nfat5^IEC-KO^* mice to assess their expression patterns. Representative staining images and the corresponding graphs are shown. Scale bars: 100 μm. (**K** and **L**) Organoids derived from small intestinal crypts from either separately housed (**K**) or cohoused (**L**) *Nfat5^fl/fl^* and *Nfat5^IEC-TG^* mice. Crypts isolated from a single mouse were seeded and cultured in Matrigel for 5 days. Representative images of organoid culture wells and the corresponding graphs are shown. Scale bars: 200 μm. Each dot in **A**–**J** represents an individual mouse, and the means are displayed as lines. Data in **K** and **L** are presented as box-and-whisker plots (minimum-to-maximum, line at the median). Data shown in **A**–**L** are representative of at least 2 independent experiments. *P < 0.05, **P < 0.01, ***P < 0.001, and ****P < 0.0001, by Mann-Whitney *U* test (**A**–**J**) and unpaired, 2-tailed Student’s *t* test (**K** and **L**).

It is widely acknowledged that the small intestine — particularly goblet cells and Paneth cells — plays a crucial role in pathogen inhibition through direct antimicrobial action. Meanwhile, the large intestine contributes to the limitation of pathogen growth by fostering competition with beneficial microbes and generating metabolic byproducts (26). Importantly, Paneth cells are absent in the colon but are primarily found in the small intestine, where they play a crucial role in regulating the gut microbiome by secreting antimicrobial peptides, such as Reg3β and 3γ, defensins, and lysozyme. The effects of NFAT5 on microbial composition prompted us to investigate how its deficiency alters this composition. To this end, we focused on 2 significant factors — mucus and antimicrobial compounds secreted by IECs, which are necessary for maintaining gut barrier function (4). We stained intestinal tissues with Alcian blue and an anti–mucin 2 antibody to detect mucin 2, the key macromolecular component of mucus, in goblet cells — the primary mucin-producing cells (27). The results showed that the colon and ileum of *Nfat5^+/–^* mice had fewer goblet cells than did those of their WT littermates (Figure 6A and Supplemental Figure 6, A and B). Concurrently, mucin 2 (*Muc2*) mRNA expression levels in IECs of the colon and ileum were lower in *Nfat5^+/–^* mice than in WT mice (Figure 6B). Next, we assessed mRNA levels of the antimicrobial compounds and found that RegIIIγ (*Reg3g*), defensin α5 (*Defa5*), and lysozyme 1 (*Lyz1*) mRNA expression levels were all significantly downregulated in the ileal epithelial cells of *Nfat5^+/–^* mice (Figure 6C). *Reg3b* mRNA expression tended to decrease, but this was not statistically significant (Figure 6C). In parallel, the number of Paneth cells (lysozyme^+^), the primary producers of antimicrobial compounds, was markedly reduced in the *Nfat5*-deficient ileum compared with that in WT controls (Figure 6D). Likewise, the number of mucin 2^+^ goblet cells and *Muc2* mRNA expression levels were lower in the IECs of *Nfat5^IEC-KO^* mice than in those of *Nfat5^fl/fl^* mice (Figure 6, E and F). The decreases in *Lyz1* mRNA expression and Paneth cell numbers were similarly reproduced in *Nfat5^IEC-KO^* mice (Figure 6, G and H). In summary, we demonstrated that *Nfat5* deficiency reduced the number of cells responsible for producing mucin and antimicrobial compounds, which may explain the gut barrier dysfunction and altered microbial composition observed in *Nfat5^+/–^* mice.

In addition to specific epithelial cell subtypes, such as goblet and Paneth cells, our findings in Figure 1, E and F, suggest that NFAT5 regulates overall IEC proliferation. ISCs continuously proliferate and differentiate to replace the old epithelium (6). We therefore suspected that the reduction in the number of cells secreting mucin and antimicrobial compounds, as well as overall IEC proliferation, stems from *Nfat5* deficiency–related dysfunction of ISCs. The data showed that *Nfat5* depletion significantly reduced the mRNA expression of ISC-related genes, such as *EphB2* and *Lgr5*, as well as the number of OLFM4^+^ cells, a marker protein of ISCs, in ileal epithelial cells (Supplemental Figure 6, C and D), indicating NFAT5-mediated regulation of ISCs. To ascertain the functional difference in ISCs between *Nfat5^+/–^* and WT mice, we established organoids from small intestinal crypts. The budding organoids derived from *Nfat5^+/–^* mice were noticeably fewer in quantity and smaller in size and budding than were those from WT mice (Supplemental Figure 6, E–G), indicative of a defect in the self-renewal and differentiation capacity of NFAT5-depleted ISCs. Intriguingly, the defect in budding organoid formation was still observed in *Nfat5*-deficient mice even after cohousing (Supplemental Figure 6H), indicating that NFAT5 controlled the regenerative capacity of IECs regardless of gut microbiota. We observed NFAT5-mediated regulation of epithelial regenerative capacity in mice with conditional deletion of epithelial *Nfat5* (Figure 6, I–L). As shown in Figure 6, I and J, the levels of *Lgr5* and *Olfm4* mRNA and the number of OLFM4^+^ cells were substantially reduced in the ileal epithelium of *Nfat5^IEC-KO^* mice. Organoids from *Nfat5^IEC-KO^* mice were also markedly fewer and smaller than were those from *Nfat5^fl/fl^* mice, regardless of gut microbe composition (Figure 6, K and L).

Collectively, our results substantiate the idea that NFAT5 plays a role in regulating the self-renewal of ISCs and their differentiation into goblet and Paneth cells, ultimately promoting the production of mucus and antimicrobial compounds. These cascades could be critical mechanisms underlying NFAT5-mediated regulation of gut microbe composition.

### HSP70 promotes the survival and proliferation of IECs and protects against DSS-induced colitis as an NFAT5 target gene.

**Figure 7:**
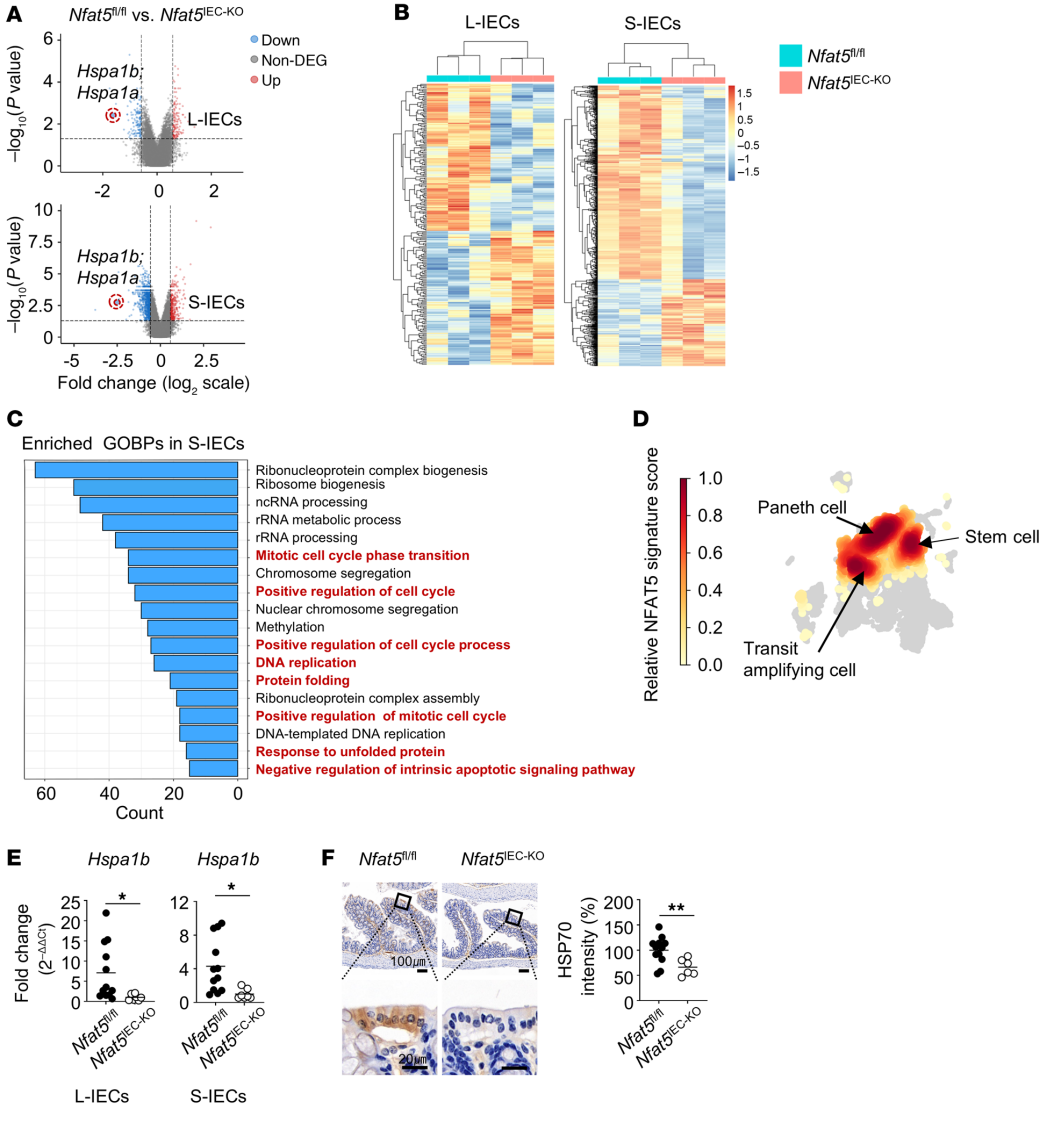
The NFAT5/HSP70 axis mediates the survival and proliferation of IECs. (**A**–**C**) Transcriptome analysis was performed using microarray on L-IECs and S-IECs isolated from *Nfat5^fl/fl^* and *Nfat5^IEC-KO^* mice (*n* = 3 per group). Volcano plots (**A**) and heatmaps (**B**) illustrate the fold change and significance and the *z* scores of DEGs, respectively. Red indicates genes upregulated in *Nfat5^IEC-TG^* versus *Nfat5^fl/fl^* IECs along with their *z* scores, and blue represents downregulated genes and their respective *z* scores. (**C**) Top 18 GOBPs enriched among downregulated DEGs in S-IECs from *Nfat5^IEC-TG^* mice compared with *Nfat5^fl/fl^* mice. Terms related to stem cells are highlighted in red. The count on the *x* axis indicates the number of enriched DEGs for each term. (**D**) Uniform manifold approximation and projection (UMAP) visualization of epithelial cell subsets with high NFAT5 signature scores (*z* score ≥0.2) in the human colonic epithelium. The color metric indicates the distribution of cells with high NFAT5 signature scores. (**E**) Relative expression levels of *Hspa1b* mRNA in L-IECs and S-IECs from *Nfat5^fl/fl^* and *Nfat5^IEC-KO^* mice were measured. *Hspa1b* expression levels were normalized to *Gapdh* mRNA. (**F**) HSP70 expression in colonic tissues from *Nfat5^fl/fl^* and *Nfat5^IEC-KO^* mice was assessed using IHC. Representative IHC images are shown, with enlarged views of the boxed areas in the lower panel. Scale bars: 100 μm and 20 μm. The corresponding graph illustrates the relative expression of HSP70 in the 2 groups. Each dot represents an individual mouse, and the mean values are displayed as lines (**E** and **F**). Data shown in **E** and **F** are representative of 2 independent experiments. *P < 0.05, **P < 0.01, ***P < 0.001, and ****P < 0.0001, by Mann-Whitney *U* test (**E** and **F**).

Our in vitro and in vivo data demonstrate that NFAT5 was essential for the survival and proliferation of IECs, although the underlying molecular mechanisms remain unclear. To understand the mechanism(s) involved, we performed microarray-based global gene expression profiling of IECs from *Nfat5* conditional-KO mice. In a comparison of *Nfat5^IEC-KO^* and *Nfat5^fl/fl^* mice, we identified 1,014 (314 upregulated and 700 downregulated genes) and 237(113 upregulated and 124 downregulated genes) differentially expressed genes (DEGs) in the small and large IECs, respectively (Figure 7, A and B). Functional enrichment analysis demonstrated that gene ontology biological process (GOBP) terms related to cell survival and proliferation, including mitotic cell-cycle-phase transition, positive regulation of the cell-cycle process, DNA replication, response to unfolded protein, and intrinsic regulation of the apoptotic pathway, were substantially enriched by DEGs (Figure 7C), which is in parallel with the data in Figure 1 and Supplemental Figure 1. To further characterize the physiological relevance of NFAT5 in IECs, we defined a set of 524 downregulated DEGs (redundant genes excluded) that were markedly enriched in the small IECs of *Nfat5^IEC-KO^* mice as the “NFAT5 signature” and compared it with the single-cell RNA-Seq (scRNA-Seq) data from human intestinal tissues, extracted from a public database (28). A total of 18 cell types were identified from the scRNA-Seq data on intestinal tissues (Supplemental Figure 7A). When the NFAT5 signature score was calculated for each cell type, we found that high signature scores (*z* score ≥0.2) were concentrated in the stem cells, Paneth cells, and transit-amplifying cells involved in epithelial repair (Figure 7D), supporting the decisive role of the NFAT5 signature in epithelial repair and regeneration.

**Figure 8:**
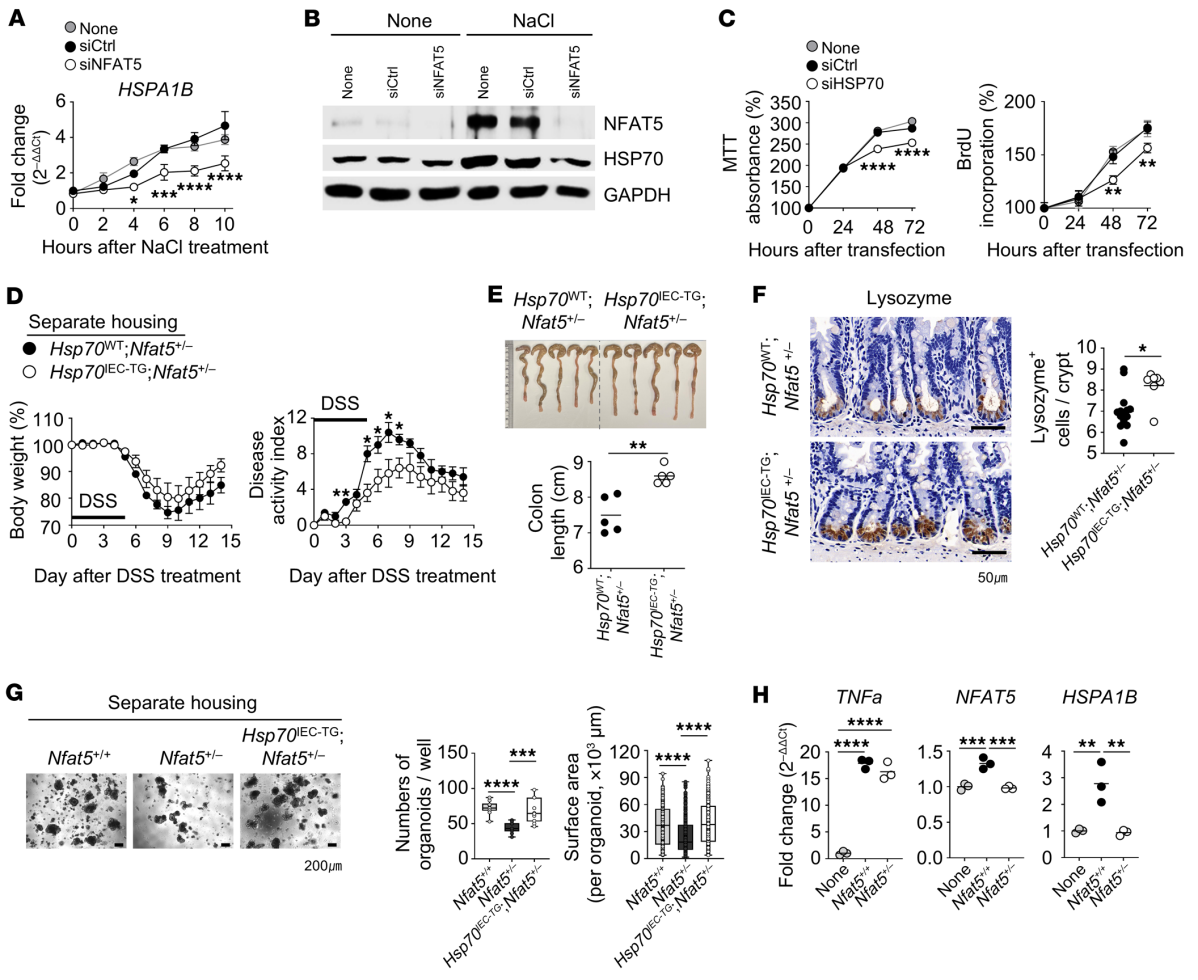
The NFAT5/HSP70 axis prevents DSS-induced colitis in mice. (**A** and **B**) Relative expression levels of *HSPA1B* mRNA (**A**) as well as of HSP70 and NFAT5 protein (**B**) were assessed in HT-29 cells exposed to 100 mM NaCl (hyperosmotic stimuli) for the specified durations (**A**) or for 24 hours (**B**) following transfection with either control siRNA (siCtrl) or *NFAT5-*targeting siRNA (siNFAT5) for 48 hours. *Gapdh* mRNA and GAPDH protein served as internal controls. (**C**) The proliferation of HT-29 cells transfected with either siCtrl or *HSP70*-targeting siRNA (siHSP70) was evaluated at the indicated time points using MTT and BrdU incorporation assays. (**D** and **E**) Body weight changes and the DAI (**D**) were monitored over 14 days following initial DSS treatment in separately housed *Hsp70^WT^* *Nfat5^+/–^* and *Hsp70^IEC-TG^* *Nfat5^+/–^* mice. Representative images of the colons and their respective lengths (**E**) on day 14 are presented. (**F**) Representative IHC images and quantification of lysozyme^+^ cells in ileal tissues from separately housed *Hsp70*^WT^ *Nfat5^+/–^* and *Hsp70^IEC-TG^* *Nfat5^+/–^* mice are shown. Scale bars: 50 μm. (**G**) 3D intestinal organoids were established by culturing small intestinal crypts isolated from separately housed *Nfat5^+/+^*, *Nfat5^+/–^*, and *Hsp70^IEC-TG^* *Nfat5^+/–^* mice in Matrigel for 5 days. Representative images of organoids, along with graphical analyses of their number and surface area, are presented. Scale bars: 200 μm. (**H**) Relative mRNA expression levels of *TNFA*, *NFAT5*, and *HSPA1B* were analyzed by qRT-PCR in HT-29 cells that were either left untreated or treated for 4 hours with fecal microbiota derived from *Nfat5^+/+^* and *Nfat5^+/–^* mice. Data are presented as the mean ± SD (**A** and **C**), the mean ± SEM (**D**), a line indicating the mean (**H**), or as box-and-whiskers plots (minimum-to-maximum, median indicated by a line (**G**). Each dot represents an individual mouse, with the mean values indicated by lines. Data shown in **A**–**H** are representative of at least 2 independent experiments. *P < 0.05, **P < 0.01, ***P < 0.001, and ****P < 0.0001, by 2-way, repeated-measures ANOVA with Šídák’s multiple-comparison test (**A** and **C** [between cells transfected with siCtrl versus siNFAT5 or siHSP70], **D**, **G**, and **H**) and Mann-Whitney *U* test (**E** and **F**).

Next, we assessed the genes that are specifically involved in the NFAT5-mediated regulation of IEC survival and proliferation. As shown in the volcano plot in Figure 7A, the *Hspa1b* gene, a member of the inducible 70 kDa HSP70 family, was the top-ranked downregulated gene. In another experiment using gene expression profiles in IECs of *Nfat5^+/–^* versus WT mice, we also found *Hspa1b* to be the top-ranked downregulated gene (Supplemental Figure 7B). HSP70 is a critical regulator of epithelial cell integrity, and aberrations in its expression can increase the severity of DSS colitis (29). Furthermore, under hypertonic conditions, NFAT5 promotes the transcription of *Hspa1b* through direct binding to its promoter (30). On the basis of our volcano plot analysis and previous reports (29, 30), we postulated that HSP70 primarily mediates the NFAT5-dependent survival, proliferation, and regenerative capacity of IECs and the progression of colitis. To prove this assumption, we first confirmed a significant decrease in *Hspa1b* mRNA and HSP70 protein expression in IECs of *Nfat5^+/–^* and *Nfat5^IEC-KO^* mice using quantitative PCR (qPCR) analysis and IHC, respectively (Figure 7, E and F, and Supplemental Figure 7, C and D). Moreover, we found that *NFAT5* knockdown using siRNAs mitigated the expression of *HSPA1B* mRNA and HSP70 protein induced by the hypertonic NaCl stimulus in HT-29 cells (Figure 8, A and B), which is consistent with the findings in a previous report (30). Notably, HSP70 knockdown using siRNA substantially reduced the survival and proliferation of HT-29 cells, as assessed by MTT and BrdU incorporation assays (Figure 8C and Supplemental Figure 8, A and B).

A previous study reported that IEC-specific *Hsp70*-Tg mice were more resistant to inflammatory colitis (29). Thus, we performed gain-of-function experiments by crossbreeding *Villin1* promoter–mediated *Hsp70*-Tg (*Hsp70^IEC-TG^*) mice with *Nfat5^+/–^* mice (Supplemental Figure 8, C and D) and challenging them with DSS. As shown in Figure 8, D and E, *Nfat5^+/–^* mice with specific overexpression of *Hsp70* in IECs (*Hsp70^IEC-TG^* *Nfat5^+/–^*) had less severe colitis than did *Nfat5^+/–^* mice without *Hsp70* overexpression (*Hsp70^WT^ Nfat5^+/–^*), demonstrating that epithelial HSP70 contributed to the attenuation of DSS-induced colitis accelerated by *Nfat5* deficiency. Moreover, the overexpression of *Hsp70* reversed the loss of Paneth cells observed in *Nfat5*-deficient mice (Figure 8F), and the decrease in organoids caused by *Nfat5* deficiency was almost completely restored by forced expression of *Hsp70* in IECs (Figure 8G), suggesting the involvement of the NFAT5/HSP70 axis in ISC self-renewal and differentiation capacity. Notably, the expression of *NFAT5* and *HSPA1B* mRNAs in HT-29 cells was induced to significant levels by adding WT feces, whereas *Nfat5^+/–^* mice feces did not show upregulation of these mRNAs (Figure 8H). Meanwhile, *Tnfa* mRNA expression as a control increased significantly with the treatment of feces from both WT and *Nfat5^+/–^* mice, with no significant difference between the 2 groups (Figure 8H). These findings suggest that the altered gut microbe composition caused by *Nfat5* deficiency renders IECs less protective, thereby explaining the protection against colitis progression in *Nfat5^+/–^* mice by FMT of WT feces shown in Figure 3J.

In summary, these data suggest that, as a direct target molecule of NFAT5, HSP70 mediated the NFAT5-dependent survival, proliferation, and regenerative capacity of IECs, consequently preventing the progression of experimental colitis.

### Epithelial NFAT5 levels and the gut microbiota contribute to the development of spontaneous colitis in mice.

**Figure 9:**
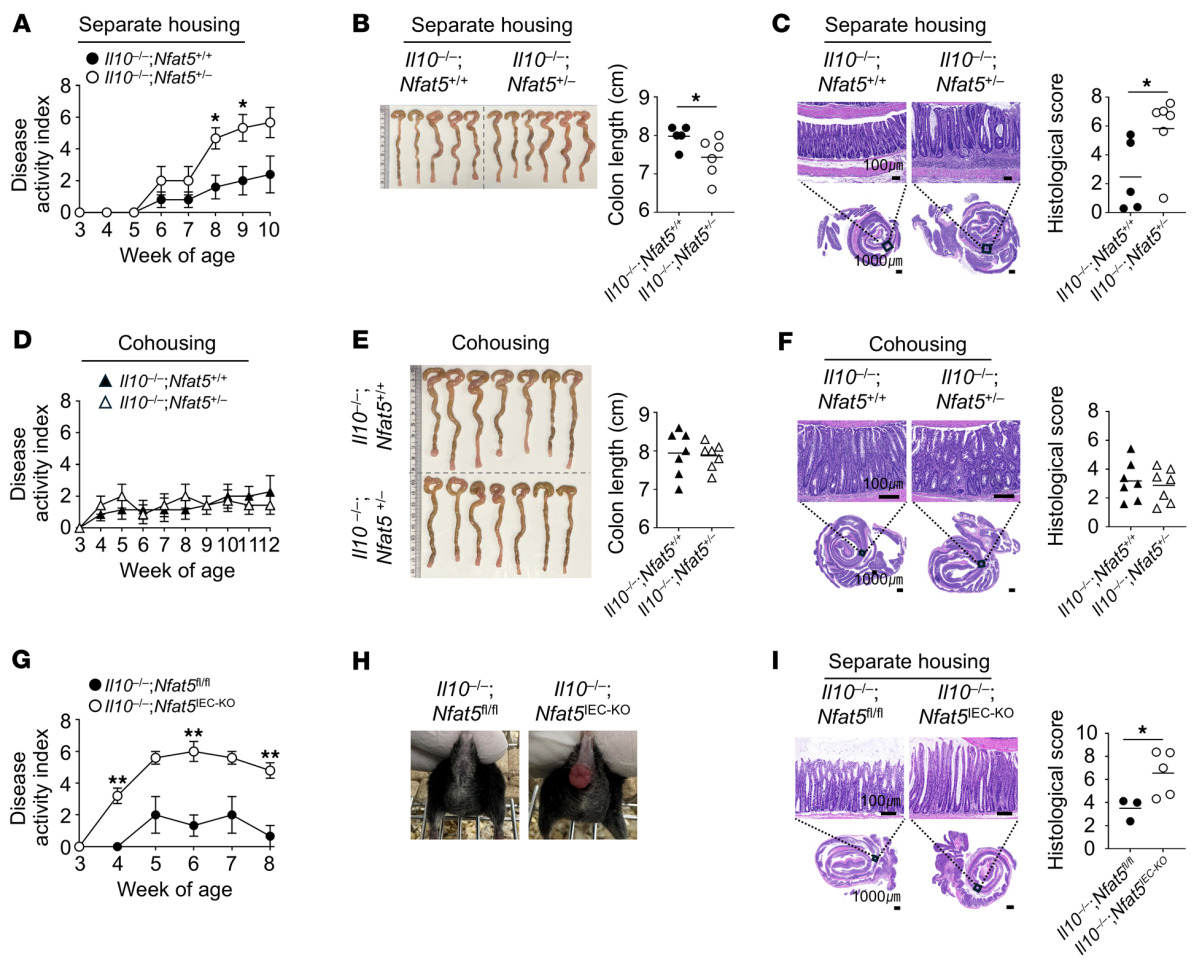
NFAT5 protects *Il10-*deficient mice from spontaneous colitis. (**A**–**F**) *Il10^–/–^* *Nfat5^+/+^* and *Il10^–/–^* *Nfat5^+/–^* mice were either housed separately by genotype (**A**–**C**) or cohoused in a mixed-genotype setting (**D**–**F**). DAI scores for separately housed (**A**) or cohoused (**D**) *Il10^–/–^* *Nfat5^+/+^* and *Il10^–/–^* *Nfat5^+/–^* mice were monitored for the indicated duration following weaning, after which their colons were collected. Monitoring of separately housed mice was stopped when mice were 10 weeks of age due to severe rectal prolapse, while the cohoused mice were monitored until 12 weeks of age. DAI scores were determined on the basis of the criteria outlined in Supplemental Table 2. Colon lengths of separately housed (**B**) and cohoused (**E**) *Il10^–/–^* *Nfat5^+/+^* and *Il10^–/–^* *Nfat5^+/–^* mice were measured and analyzed. Representative H&E images and corresponding histological score graphs of separately housed (**C**) or cohoused (**F**) *Il10^–/–^* *Nfat5^+/+^* and *Il10^–/–^* *Nfat5^+/–^* mice are presented. Histological scoring was conducted on the basis of the criteria in Supplemental Table 4. (**G**–**I**) *Il10^–/–^* *Nfat5^fl/fl^* and *Il10^–/–^* *Nfat5^IEC-KO^* mice were housed separately by genotype. DAI scores were monitored until 8 weeks of age, at which point monitoring was stopped due to severe rectal prolapse (**G** and **H**). Representative H&E images of colons and corresponding histological score graphs are presented (**I**). Scale bars: 100 μm and 1,000 μm (**C** and **I**). Data in **A**, **D**, and **G** represent the mean ± SEM. Data points in **B**, **C**, **E**, **F**, and **I** represent individual mice, and group means are depicted as horizontal bars. Data shown in **A**–**G** and **I** are representative of at least 2 independent experiments. *P < 0.05 and **P < 0.01, by 2-way, repeated-measures ANOVA with Šídák’s multiple-comparison test (**A**, **D**, and **G**) and Mann-Whitney *U* test (**B**, **C**, **E**, **F**, and **I**).

Although DSS-induced colitis is widely used in experimental colitis, it does not involve T or B cells, unlike human IBD (31). In contrast, *Il10*-deficient mice develop spontaneous colitis with increased Th1 and Th17 responses and abundant production of proinflammatory cytokines, mimicking human IBD pathogenesis (32). To confirm the main findings of this study in another model of experimental colitis that more closely resembles the development of human IBD, we mated *Il10*-homozygous KO (*Il10^–/–^*) mice with *Nfat5*-heterozygous KO (*Nfat5^+/–^*) mice (Supplemental Figure 9) and compared the development of spontaneous colitis between the *Nfat5^+/–^* and *Nfat5^+/+^* littermates. The experiments ended before any difference in body weight was observed (data not shown) because severe rectal prolapse was observed in some *Il10* and *Nfat5* double-KO (*Il10^–/–^* *Nfat5^+/–^*) mice. Nonetheless, compared with the *Il10^–/–^* *Nfat5^+/+^* mice, the *Il10^–/–^* *Nfat5^+/–^* mice had higher DAI scores, more frequent rectal prolapse, and more severe shortening and microscopic pathology of the colon (Figure 9, A–C). Importantly, these differences disappeared when the mice were cohoused (Figure 9, D–F), which concurs with the cohousing data observed in the DSS-induced colitis model (Supplemental Figure 2), suggesting that the gut microbiota is critical for NFAT5-dependent protection against inflammatory colitis in this spontaneous model as well.

**Figure 10:**
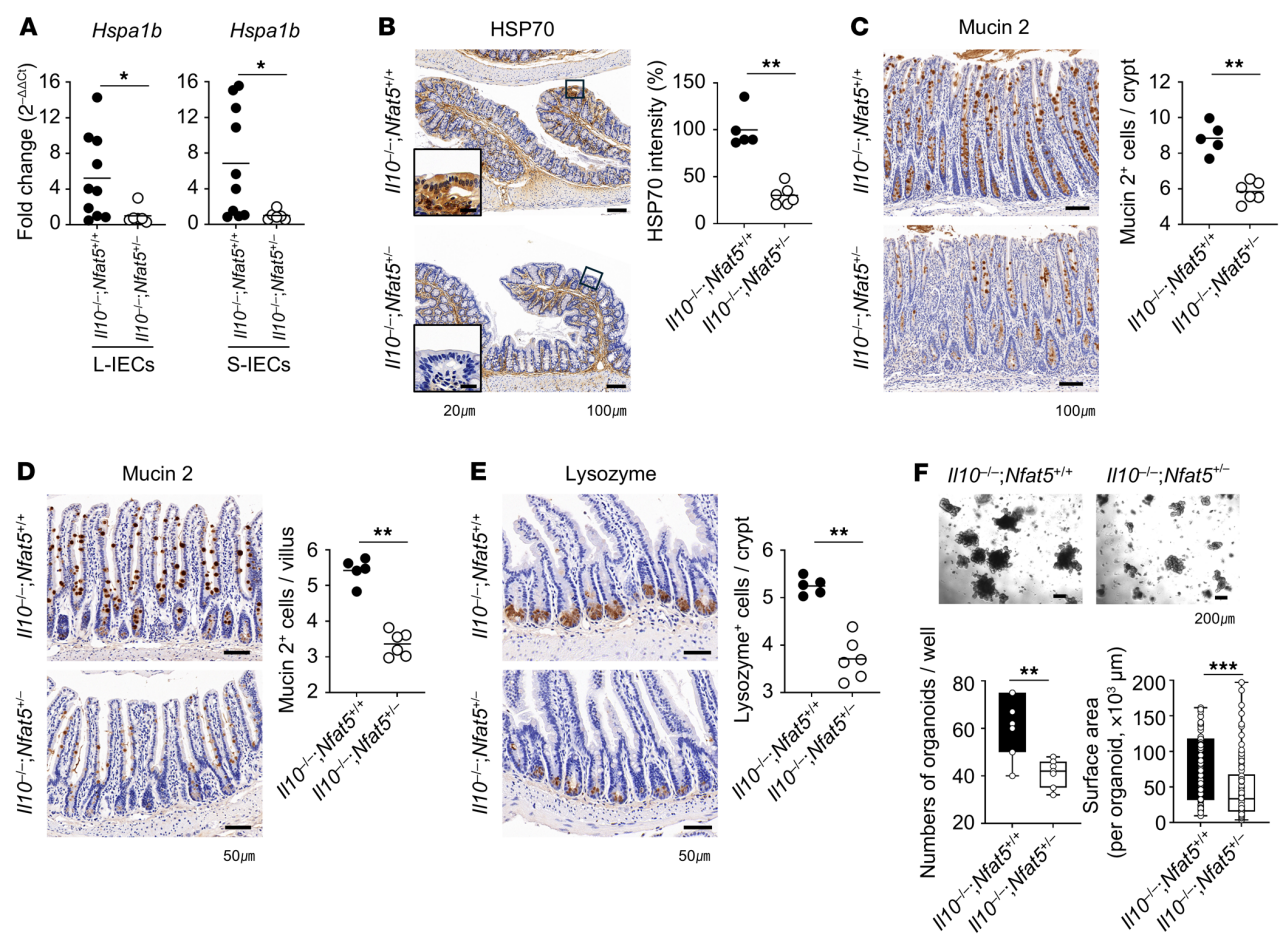
NFAT5 deficiency exacerbates spontaneous colitis in *Il10*-deficient mice by influencing gut microbiota composition and impairing epithelial regenerative capacity. (**A**) Relative expression levels of *Hspa1b* mRNA in L-IECs and S-IECs from separately housed 10-week-old *Il10^–/–^* *Nfat5^+/+^* and *Il10^–/–^* *Nfat5^+/–^* mice were quantified by qRT-PCR and normalized to *Gapdh* mRNA. (**B**–**E**) IHC staining for HSP70 (**B**) and mucin 2 (**C**) in colonic tissues, as well as mucin 2 (**D**) and lysozyme (**E**) in ileal tissues, was carried out for separately housed *Il10^–/–^* *Nfat5^+/+^* and *Il10^–/–^* *Nfat5^+/–^* mice at 10 weeks of age. Representative images are and the corresponding graphs are shown. (**F**) Organoids were generated by culturing intestinal crypts isolated from separately housed *Il10^–/–^* *Nfat5^+/+^* and *Il10^–/–^* *Nfat5^+/–^* mice in Matrigel for 5 days. Crypts were isolated from the small intestine of individual mice, and the efficiency of organoid formation was assessed by measuring both the number and surface area of organoids generated per well. Multiple images were acquired from each well, and the surface area of all organoids within these images was quantified. Representative images of the formed organoids are shown. Each dot represents an individual mouse, with group means indicated by horizontal lines (**A**–**E**). Data shown in **A**–**E** are representative of at least 2 independent experiments; data in **F** are representative of 3 independent experiments and are presented as box-and-whiskers plots (minimum-to-maximum, line at median). *P < 0.05 and **P < 0.01, and ***P < 0.001 by Mann-Whitney *U* test (**A**–**E**) and unpaired, 2-tailed Student’s *t* test (**F**). Scale bars: 100 μm and 1,000 μm (**C**, **F**, and **I**).

To further determine the role of epithelial NFAT5 in spontaneous colitis, we generated conditional-KO *Il10^–/–^* *Nfat5^IEC-KO^* and their *Il10^–/–^* *Nfat5^fl/fl^* mice. As expected, these mice, with conditional deletion of *Nfat5* in the IECs exhibited more severe colitis with higher DAI and histological scores and rectal prolapse (Figure 9, G–I). Despite being IL-10 deficient, *Nfat5* depletion suppressed the expression levels of *Hspa1b* mRNA and HSP70 protein and diminished the number of goblet (mucin 2^+^) and Paneth (lysozyme^+^) cells (Figure 10, A–E). Moreover, budding organoids from *Il10^–/–^* *Nfat5^+/–^* mice were significantly fewer in number and smaller in size than were those from *Il10^–/–^* *Nfat5^+/+^* mice (Figure 10F), confirming NFAT5-dependent regulation of IEC regeneration.

Collectively, these results demonstrate that *Nfat5* deficiency played a crucial role in the development of IL-10–dependent spontaneous colitis. This association appeared to stem from mechanisms involving the dysregulated NFAT5/HSP70 axis and changes in the gut microbiota, similar to those observed in DSS-induced colitis.

## Discussion

This study is the first to our knowledge to demonstrate that the protective role of NFAT5 in colitis is mediated via its function in IECs. Although NFAT5 is widely recognized as an osmo-adaptive transcription factor critical for immune and renal medullary cells (33), its role in the intestinal epithelium was previously unclear. Our findings address this knowledge gap by revealing that epithelial NFAT5 was beneficial for the regeneration of ISCs and formation of the mucosal barrier. Specifically, we observed that NFAT5 functioned as a key mediator in the crosstalk between IECs and the gut microbiome, facilitating a favorable microbial composition that prevented excessive inflammation and enhanced the gut barrier. We believe this NFAT5-driven axis (intestinal stem cells→microbial homeostasis→colitis protection) substantially enhances our understanding of how intrinsic or extrinsic epithelial factors contribute to intestinal immune homeostasis.

NFAT5 has been increasingly implicated in cell survival, proliferation, and migration across various contexts (15, 16, 20, 34). In this study, NFAT5 deficiency meaningfully impaired the proliferation and survival of IECs, dampening the self-renewal and differentiation capacity of ISCs and the formation of intestinal organoids. These intrinsic characteristics associated with NFAT5 deficiency may partially underlie the increased susceptibility to chemically induced or spontaneous colitis observed in *Nfat5*-deficient mice. In particular, epithelial NFAT5 was found to orchestrate the mucosal microenvironment by regulating mucus production and antimicrobial peptides. Secreted gel-forming mucins, largely mucin 2, form a polymeric net-like mucus layer outside the intestinal epithelium. Mucins can directly influence the gut microbial composition, as microorganisms degrade and utilize mucin glycans for both energy and attachment (35). In our microbiome data, the diminished prevalence of the genus *Ruminococcus*, categorized as a mucin-degrading microbe (36), may be attributed to lower mucin 2 production in *Nfat5*-deficient mice. Along with mucin production, gradients of antimicrobial compounds are formed in the mucus layer, thereby preventing the infiltration of most bacteria (37, 38). Despite the commonly perceived limited microbicidal effect of antimicrobial compounds, their concentrations in intestinal crypts can reach levels capable of robust bacterial lysis, thereby influencing the gut microbe composition (38). These mechanisms may explain how NFAT5 shapes the gut microbe composition.

The intestinal epithelial barrier, composed of IECs interconnected by tight junctions, is required for maintaining intestinal homeostasis, which enables the establishment of a balanced environment permissive for colonization by commensal bacteria (4). In contrast, impairment of barrier integrity, commonly referred to as “leaky gut,” initiates pathological alterations linked to the development of IBD (39). Certain commensal bacteria can promote the expression of tight-junction molecules in IECs via their components and metabolites (4, 39, 40). Among these commensals, a subset regulated by NFAT5 may specifically contribute to modulating tight-junction integrity. For example, feces from *Nfat5*-deficient mice contained fewer microbes from the genera *Lachnospiraceae*_NK4A136_group and *Faecalibacterium*, which are important butyrate producers with diverse effects on intestinal health, such as the maintenance of intestinal barrier function and antiinflammation (40–42).

In addition to distinct microbiome differences associated with NFAT5 expression, our findings from separate housing and cohousing experiments suggest that the gut microbiota influenced colitis severity in *Nfat5-*deficient mice. Moreover, FMT experiments under antibiotic-driven, germ-free conditions clearly demonstrated that gut microbiota, compromised by *Nfat5* deficiency, directly contributed to the progression of DSS-induced colitis. However, the inherent limitations of 16S rRNA amplicon sequencing preclude the identification of specific microorganisms. Furthermore, we could not identify any culturable bacteria at the species level and therefore failed to pinpoint specific pathobionts that are directly responsible for exacerbating colitis in the context of *Nfat5* deficiency. Further studies utilizing shotgun metagenomic sequencing will allow for the precise identification of microbiota altered by NFAT5 expression at the species or strain level, facilitating the determination of whether specific microbe changes represent a causative factor or a consequence of the phenotype observed in *Nfat5*-deficient mice.

Our study carries translational relevance by linking epithelial NFAT5 to IBD. Previous studies have reported reduced NFAT5 expression in rectal biopsies from patients with active ulcerative colitis and Crohn’s disease (43). Based on our study, reduced NFAT5 expression may impair mucosal healing and compromise barrier integrity, thereby exacerbating IBD. This idea is further supported by a reported case involving an immunodeficient patient with only 1 functional copy of *NFAT5*, who developed severe autoimmune enterocolopathy and chronic bowel inflammation (43), suggesting the involvement of NFAT5 in gut immune homeostasis in humans. In contrast to previous studies that focused on NFAT5 in immune cells (16, 19, 20), our findings reveal that epithelial NFAT5 serves as a critical safeguard for gut integrity. Specifically, NFAT5 loss might disrupt IEC-mediated microbiome regulation and mucosal immunity, creating a permissive environment for inflammation in patients with IBD. Our findings not only corroborate these earlier studies but also extend them by pinpointing a specific NFAT5-dependent pathway in IECs that underlies IBD pathogenesis.

Notably, despite its detrimental effects, NFAT5 deficiency did not appear to entirely abolish IEC formation. NFAT5-deficient mice retained an epithelial layer and some functional stem cells, although their regeneration was much slower under both normal and stress conditions. These findings indicate that NFAT5 serves as a modulatory, rather than essential, factor in epithelium development, maintenance of gut homeostasis, and stress responses. Basic proliferation and differentiation can proceed without NFAT5, likely due to compensatory pathways. However, its absence may increase vulnerability to inflammation and osmotic stress. This explains the mild baseline phenotype and its worsening under excessive stress, highlighting the role of NFAT5 in reinforcing epithelium resilience. In this regard, evaluating NFAT5 levels could be clinically useful for the early detection of epithelium resilience to stress. The preserved yet dysfunctional IECs suggest that therapeutic restoration is feasible without harming normal architecture.

Our work also identifies the NFAT5/HSP70 axis as a potential protective mechanism for ISCs. Transcriptomics analyses identified *Hspa1b*, which encodes HSP70, as a key downstream target of NFAT5 in IECs. This finding was further validated by demonstrating NFAT5-driven HSP70 expressions in gut epithelial cells. Notably, NFAT5 has been shown to induce HSP70 transcription in other biological contexts as well (44). Our results point to the relevance of the NFAT5/HSP70 pathway to maintaining ISC renewal and differentiation. HSP70, a cytoprotective chaperone, protects cells from injury; thus, NFAT5-mediated HSP70 upregulation appears to help IECs withstand inflammation-induced damage. This concept aligns with previous findings that transgenic mice with IEC-specific HSP70 overexpression show attenuated progression of DSS-induced colitis (29). Consequently, NFAT5 regulation of HSP70 presents a plausible mechanism for intestinal barrier preservation, whereby NFAT5-enhanced HSP70 expression enables intestinal stem and progenitor cells to manage stress from inflammatory or osmotic challenges, thereby supporting continuous renewal of the epithelium. Therapeutically, enhancement of the NFAT5/HSP70 axis might reinforce intestinal barrier integrity and reduce IBD severity, although careful evaluation of such interventions is necessary.

The present study opens new avenues for further research. First, the molecular details of the NFAT5/HSP70 axis in ISC biology should be further elucidated. In particular, it seems important to determine how the NFAT5/HSP70 axis regulates the stemness and differentiation of ISCs. Investigating other NFAT5 target genes in IECs could provide insights into this question. NFAT5 may regulate a broad network of other heat shock proteins, trefoil factors, tight-junction proteins, and Wnt pathway modulators that collectively promote epithelium integrity and maintain the stem cell niche (4, 7). Another key question is how NFAT5 activity in IECs is coordinated with mechanical circumstances affected by diet, microbiota, and immune status. Since NFAT5 can be activated by gut osmolarity, which changes with diet or inflammation (18), it is plausible that dietary factors (e.g., high salt or fiber content) modulate NFAT5 expression in the gut and thereby influence disease outcomes. In short, future mechanistic studies should aim to delineate the NFAT5-centered regulatory network that preserves epithelial homeostasis, potentially uncovering additional targets or strategies to strengthen this natural protective mechanism in the intestine.

In summary, this study identifies NFAT5 as a previously unrecognized intrinsic protector of IECs that is crucial for both injury regeneration and the maintenance of a protective microbiome under stress. NFAT5 deficiency unveils a 2-pronged mechanism of colitis susceptibility: an epithelium-intrinsic vulnerability to injury and an extrinsic shift in the microbiota toward a colitogenic profile. This contrasts with traditional T cell–mediated colitis and expands our understanding of how epithelial stress adaptation factors contribute to inflammatory diseases. Our findings also have potential clinical ramifications. Given that NFAT5 expression is diminished in patients with active IBD (43), approaches aimed at enhancing epithelial NFAT5 activity or its downstream pathways may reinforce gut barrier integrity, improving healing in an inflammatory setting. Additionally, microbiome alterations caused by NFAT5 deficiency underscore the importance of precision microbiome-based therapies. Specifically, the targeted restoration of beneficial commensals or sustained modulation of the microbiota could synergize with pharmacological approaches to strengthen epithelium resilience. Overall, our work underscores a model in which both the host epithelium and its microbiome are integral to IBD pathogenesis, paving the way for precision medicine approaches that address the host-microbiome crosstalk to achieve lasting remission in IBD.

## Methods

### Sex as a biological variable.

Although both male and female animals exhibited similar findings, only male mice were used in subsequent experiments, as they developed more pronounced and aggressive symptoms of DSS-induced colitis than did female mice.

### Animals.

All mice used in this study were 9- to 12-week-old male C57BL/6J background mice and were maintained under specific pathogen–free (SPF) conditions in an animal facility at The Catholic University of Korea and SNU. *Nfat5^+/–^* and *Nfat5^fl/fl^* mice were provided by Hyug Moo Kwon (Ulsan National Institute of Science and Technology, Ulsan, South Korea). *Nfat5^IEC-TG^* mice were generated by crossing *Nfat5^fl/fl^* mice with *Vil-Cre* *1000* mice (obtained from The Jackson Laboratory). *Il10^–/–^* *Nfat5^+/–^* mice were generated by crossing *Nfat5^+/–^* mice with *Il10*^–/–^ mice (a gift of Sang-Uk Seo, The Catholic University of Korea, Seoul, South Korea). *Hsp70^IEC-TG^* *Nfat5^+/–^* mice were generated by crossing *Nfat5^+/–^* mice with *Villin-Hsp70*–Tg mice (provided by Eugene B. Chang, University of Chicago IBD Research Center, Chicago, Illinois, USA). Mice were separated or cohoused for at least 6 weeks after weaning.

### DSS-induced colitis.

The mice were given 2% (w/v) DSS (colitis grade, M.W. 36–50 kDa; MP Biomedicals, 160110) in the drinking water ad libitum for 5 days, followed by 9 days of normal drinking water. DAI, ranging from 0–13, was determined by scoring weight loss (<1%, 0; 1%–5%, 1; 5%–10%, 2; 10%–15%, 3; 15%–20%, 4; >20%, 5), stool consistency (normal, 0; loose stool, 2; diarrhea, 4), and rectal bleeding (no bleeding, 0; hemoccult^+^, 2; gross bleeding or blood around the anus, 4). On day 14, the mice were euthanized, and the colon length was measured. The histopathologic score was calculated by scoring inflammatory cell infiltration and intestinal architecture, as detailed in Supplemental Table 3.

### Il10^–/–^ spontaneous colitis.

The mice were monitored for 5–9 weeks after weaning. The DAI, ranging from 0–12, was determined by scoring stool consistency (normal stool, 0; loose stool, 2; diarrhea, 4), rectal bleeding (no bleeding, 0; hemoccult^+^, 2; gross bleeding or blood around the anus, 4), and rectal prolapse (no prolapse, 0; prolapse evident only during defecation, 2; prolapse evident at all times, 4). At 8–12 weeks of age, the mice were euthanized, and the colon length was measured. The histopathologic score was calculated by scoring inflammatory cell infiltration and epithelial changes, such as hyperplasia and abscess of the crypt, as detailed in Supplemental Table 4.

### FMT.

To facilitate the intestinal colonization of donor microbes introduced by FMT, recipient mice were provided with an antibiotic cocktail containing ampicillin (1 g/L) (MilliporeSigma, A0166), metronidazole (1 g/L; MilliporeSigma, M1547), neomycin (1 g/L; MilliporeSigma, N5285), and vancomycin (0.5 g/L; MilliporeSigma, PHR1732), supplemented with sucrose (50 g/L; MilliporeSigma, 84097) in distilled water for 3 weeks before the FMT. Feces were freshly collected from separately housed donor mice, dissolved in drinking water (1 g/3 mL), filtered through a 100 μm pore strainer (Falcon, 352360), and administered to each mouse by oral gavage (100 mg/300 μL) every other day, 3 or 5 times a day. Following the last day of FMT, the recipient mice were provided 2% (w/v) DSS in the drinking water ad libitum for 5 days, followed by 9 days of normal drinking water.

### FITC-dextran intestinal permeability assay.

After a 12-hour fast from food and water, mice were orally gavaged with 200 μL FITC-dextran (0.6 mg/g; MilliporeSigma, 46944, average molecular weight 4,000, FITC/glucose = 1:250), and their blood was collected by cardiac puncture 4 hours later. The serum sample was obtained by centrifugation at 3,000 rpm for 30 minutes at 4°C. The concentration of the FITC-dextran in the sera was determined by a fluorometer with an excitation wavelength of 490 nm and an emission wavelength of 530 nm. Serially diluted FITC-dextran was used as a standard.

### Statistics.

Statistical analyses were performed using GraphPad Prism 10.2.0 (GraphPad Software). Data were tested for normality with the Shapiro-Wilk test. Comparisons between 2 groups were made with an unpaired, 2-tailed Student’s *t* or Mann-Whitney *U* test. Comparisons among multiple groups and time-course data were performed using a 2-way ANOVA with Šídák’s multiple-comparison test. *P* values of less than 0.05 were considered significant. Data are presented as the mean ± SEM or ± SD, as indicated in the figure legends.

### Study approval.

All animal experiments were performed in accordance with protocols approved by the IACUC of SNU (SNU-191017-15-5, SNU-220121-1-1, SNU-221118-2) and the Catholic University of Korea in Seoul (CUMS-2018-0052-04, CUMS-2022-0051-01, CUMS-2023-0037-01).

### Data availability.

All supporting data are provided in the Supporting Data Values file. All raw 16S rRNA amplicon sequences derived from this experiment were deposited in the NCBI’s Short Read Archive (SRA) and can be found under the BioProject accession number PRJNA1065788. The microarray data are available in the NCBI’s Gene Expression Omnibus (GEO) database (GEO GSE256421). Any additional information required to reanalyze the data reported in this article is available from the corresponding author upon request.

Other detailed experimental methods, including cell lines and cell cultures, siRNA transfection, MTT assay, trypan blue dye exclusion assay, BrdU colorimetric assay, annexin V and propidium iodide (PI) staining, immunocytochemistry, histology and IHC, immunofluorescence staining, in vivo EdU incorporation assay, RNA extraction and quantitative real-time qRT-PCR, isolation of intestinal epithelial cells, microarray analysis, scRNA-Seq analysis based on public database, Western blotting, DNA extraction and next-generation sequencing of 16S rRNA, bioinformatics analysis, and crypt isolation and small intestinal organoid cultures are described in the Supplemental Methods.

## Author contributions

WUK and DK were responsible for study conceptualization. SHP, DHC, YMK, YJC, SHC, MNK, EBC, HMK, DK, and WUK designed the study methodology. SHP, DHC, YMK, YC, and DK performed experiments. SHP, DHC, BKH, DK, and WUK performed visualization. Funding acquisition: YMK, DK, and WUK acquired funding. SHP, DHC, DK, and WUK were responsible for project administration. DK and WUK supervised the study. SHP, DHC, BKH, DK, and WUK wrote the original draft of the manuscript. All authors reviewed and edited the manuscript.

## Supplementary Material

Supplemental data

Unedited blot and gel images

Supporting data values

## Version 1 07/15/2025

In-Press Preview

## Version 2 09/16/2025

Electronic publication
