# Supplementary material for: NFAT5 dictates crosstalk between intestinal epithelial regenerative capacity and microbiota in murine colitis models
Source: J Clin Invest. 2025 Jul 15;135(18):e183093. doi: 10.1172/JCI183093 (PMC12435833; doi:10.1172/JCI183093)
Supplement: Supplemental data [file jci-135-183093-s142.pdf]

Supplemental Data Information
NFAT5 dictates crosstalk between intestinal epithelial regenerative capacity and microbiota in murine colitis models

Se-Hyeon Park,[1,2,a] Dae Hee Cheon,[3,4,a] Yu-Mi Kim,[1,2] Yeji Choi,[3,4]
Yong-Joon Cho,[5,6] Bong-Ki Hong,[1,2] Sang-Hyun Cho,[7] Mi-Na Kweon,[8] Hyug Moo Kwon,[9]
Eugene B. Chang,[10] Donghyun Kim,[3,4,11,12,b] and Wan-Uk Kim[1,2,13,b]

[1]*Center for Integrative Rheumatoid Transcriptomics and Dynamics, The Catholic University of Korea, Seoul, Republic of Korea*
[2]*Department of Biomedicine & Health Sciences, The Catholic University of Korea, Seoul, Republic of Korea*
[3]*Department of Biomedical Sciences, Seoul National University College of Medicine, Seoul, Republic of Korea*
[4]*Department of Microbiology and Immunology, Seoul National University College of Medicine, Seoul, Republic of Korea*
[5]*Department of Molecular Bioscience, Kangwon National University, Chuncheon, Republic of Korea*
[6]*Multidimensional Genomics Research Center, Kangwon National University, Chuncheon, Republic of Korea*
[7]*School of Biological Sciences, Seoul National University, Seoul, Republic of Korea*
[8]*Mucosal Immunology Laboratory, Department of Convergence Medicine, University of Ulsan College of Medicine/Asan Medical Center, Seoul, Republic of Korea*
[9]*Department of Biological Sciences, Ulsan National Institute of Science and Technology, Ulsan, Republic of Korea*
[10]*Department of Medicine, University of Chicago IBD Research Center, Chicago, Illinois 60637, USA*
[11]*Institute of Endemic Diseases, Seoul National University Medical Research Center, Seoul, Republic of Korea*
[12]*Institute of Cancer Research, Seoul National University, Seoul, Republic of Korea*
[13]*Division of Rheumatology, Department of Internal Medicine, the Catholic University of Korea, Seoul, Republic of Korea*

[a]These authors contributed equally to this work
[b]Correspondence and reprint requests to: Dr. Donghyun Kim, Rm#314, Research Bldg, 103 Daehak-ro, Jongno-gu, Seoul, Republic of Korea, 03080, +82-02-740-8304, biologokim@snu.ac.kr or Dr. Wan-Uk Kim, Rm#1209, 222, Banpo-daero, Seocho-gu, Seoul, Republic of Korea, 06591, +82-02-3147-8725, wan725@catholic.ac.kr.

Keywords: NFAT5, gut epithelial renewal, gut microbiome, heat shock protein 70, inflammatory bowel disease

This file includes:

Supplemental Methods

Supplemental Figure 1. Effect of NFAT5 on the proliferation and survival of IECs.

Supplemental Figure 2. Differential impact of separate housing versus cohousing on the severity of DSS-induced colitis in *Nfat5*$^{+/+}$ *and Nfat5*$^{+/–}$ mice.

Supplemental Figure 3. Selective deletion of NFAT5 in IECs of *Nfat5*$^{\text{IEC-KO}}$ mice and its effects on epithelial cell proliferation and apoptosis.

Supplemental Figure 4. Expression of Occludin (Ocln), a tight junction-associated protein, is reduced by *Nfat5* deficiency and exposure to feces of *Nfat5*$^{+/–}$ mice.

Supplemental Figure 5. Changes in the composition of cecal microbiota by NFAT5 deficiency.

Supplemental Figure 6. NFAT5 affects the production of mucin from the goblet cells through the regulation of epithelial regenerative capacity.

Supplemental Figure 7. Transcriptomic profiling of human and mouse IECs reveals HSP70 as a major NFAT5-regulated gene.

Supplemental Figure 8. HSP70 expression levels in *Hsp70*-knockdown cells and *Hsp70*$^{\text{IEC-TG}}$ mice.

Supplemental Figure 9. NFAT5 expression in IECs of *Il10*$^{–/–}$; *Nfat5*$^{+/+}$ and *Il10*$^{–/–}$; *Nfat5*$^{+/–}$ mice.

Graphical Abstract: Hypothetical model for the role of NFAT5 in maintaining gut homeostasis and protecting against colitis.

Supplemental Table 1. Disease Activity Index scoring for the DSS-induced colitis model.

Supplemental Table 2. Disease Activity Index scoring for the *IL-10*$^{–/–}$ spontaneous colitis model.

Supplemental Table 3. Histopathological scoring for the DSS-induced colitis model.

Supplemental Table 4. Histopathological scoring for the *IL-10*$^{–/–}$ spontaneous colitis model.

Supplemental Table 5. Primer sequences for real-time PCR

Supplemental References

Supplemental Methods

Cell lines and cell culture

The human colon adenocarcinoma cell line HT-29 and Caco-2 cells were obtained from the Korean Cell Line Bank. HT-29 cells were cultured in RPMI 1640 (Welgene, LM011-03) supplemented with 10% FBS (Gibco, 12483020) and 1x antibiotic-antimycotic (Gibco, 15240-062). Caco-2 cells were maintained in Minimum Essential Medium (MEM) (Gibco, 11095080) supplemented with 20% FBS (Gibco), 100mM sodium pyruvate (Gibco, 11360070), 1x non-essential amino acids solution (Gibco, 1114050), 1x β-mercaptoethanol (Gibco, 21985-023), and 1x antibiotic-antimycotic (Gibco, 15240-062). Cells were cultured at 37°C in a 5% CO_2 atmosphere. Transfection experiments were performed in the absence of 1x antibiotic-antimycotic (Gibco).

siRNA transfection

HT-29 and Caco-2 cells were seeded at $1x10^5$ cells/well and $4x10^4$ cells/well in a 24-well plate, respectively. After 24 hours, HT-29 cells were transfected with 50 nM Nontargeting pool (Dharmacon, D-001910-10-20) or ON-TARGETplus SMARTpool siRNA for human *NFAT5* (Dharmacon, L-009618-00-0020), and 30 nM control (Santa Cruz, sc-37007) or *HSP70* siRNA (Santa Cruz, sc-29352), and Caco-2 cells were transfected with 100 nM control (Santa Cruz, sc-37007) or *NFAT5* siRNA (Santa Cruz, sc-43968) using Lipofectamine 3000 (Invitrogen, L3000-015) according to the manufacturer's instructions.

MTT (3-[4,5-dimethylthiazol-2-yl]−2,5-diphenyltetrazolium bromide) assay

After transfection with *NFAT5, HSP70*, or control siRNAs for 0, 24, 48, and 72 hours, HT-29 and Caco-2 cells were treated with MTT (0.5 mg/mL; Sigma-Aldrich, M2128)

dissolved in complete growth medium at 37°C in a 5% CO_2 atmosphere in the dark for 1 hour. The medium was removed and formazan crystals were dissolved using DMSO (Junsei, 35535S0350) before being transferred to a 96-well plate. The absorbance was measured at 570 nm.

Trypan blue dye exclusion assay

HT-29 and Caco-2 cells were detached by adding 0.25% Trypsin-EDTA (Gibco, 25200-056) after transfection with control or *NFAT5* siRNAs for 0, 24, 48, and 72 hours. The cell suspensions were mixed with 0.4% Trypan Blue (Gibco, 15250-061). The viable cells were counted using a hemocytometer under a light microscope.

BrdU colorimetric assay

Following transfection with control, *NFAT5, or HSP70* siRNAs for 0, 24, 48, and 72 hours, HT-29 and Caco-2 cells were subjected to a cell proliferation assay using a BrdU colorimetric kit (Roche Diagnostics, 11647229001) according to the manufacturer's instructions.

Annexin V and propidium iodide (PI) staining

HT-29 and Caco-2 cells were transfected with control or *NFAT5* siRNAs for 48 hours. The medium was removed, and the cells were treated with 5 μg/mL tunicamycin (Sigma-Aldrich, T7765), 100 nM thapsigargin (Sigma-Aldrich, T9033), and 100 mM butyrate (Sigma-Aldrich, B5887) for 24 hours at 37°C in a 5% CO_2 atmosphere. After DPBS washing, cell apoptosis was determined with FITC-Annexin V Apoptosis Detection Kit I (BD Biosciences, 556547). Annexin V staining was carried out according to the manufacturer's instructions.

Acquisition was conducted on a BD CantoII Flow Cytometer (BD Biosciences), and analysis was carried out with FlowJo software (v10.7.1).

Immunocytochemistry

HT-29 and Caco-2 cells were fixed with 4% (w/v) paraformaldehyde in PBS (Wako) for 10 minutes at room temperature (RT). After permeabilization with 0.5% Triton X-100 (Sigma-Aldrich, T8787) for 15 minutes at RT, the cells were blocked with normal donkey serum (Jackson ImmunoResearch, 017-000-121) diluted in PBS (1:10) for 1 hour and incubated with an anti-Ki-67 antibody (1:3000, Abcam, ab16667, clone SP6) overnight at 4°C. The slides were then treated with a Biotin-SP AffiniPure Donkey Anti-Rabbit IgG (H+L) (Jackson ImmunoResearch, 711-065-152) diluted in PBS (1:200) for 2 hours. After washing in DPBS, a VECTASTAIN ABC kit (Vector laboratories, PK-6100) was added and incubated for 1 hour. The cells were stained with DAB substrate kit (Vector Laboratories, SK-4100), and the nuclei were counterstained using Harris hematoxylin (YD Diagnostics, S2-5). Cells were dehydrated in ethanol (70 to 100%) and mounted using MM24 (Leica). The slide images were acquired using a Pannoramic SCAN II scanner (3DHISTECH Ltd).

Histology and immunohistochemistry

The ileum and colon were removed, longitudinally cut open, washed with ice-cold PBS, rolled up, and fixed with 4% (w/v) paraformaldehyde in PBS (Biosesang, P2184) overnight at 4 °C. After washing with PBS, the tissues were embedded in paraffin and sectioned at a thickness of 4 μm. The sections were deparaffinized in xylene and stained with hematoxylin and eosin (H&E). For Alcian blue/Fast red staining, the tissues were rehydrated

with ethanol (100 to 70%) and incubated in Alcian blue solution (Sigma-Aldrich, B8438) for 10 minutes at RT. The nuclei were counterstained using Nuclear fast red-aluminum sulfate solution (Merck Millipore, 100121) for 5 minutes.

For immunohistochemistry, the sections were deparaffinized in xylene and rehydrated with ethanol (100 to 70%). Antigen retrieval was performed in citrate buffer (pH 6.0) (Sigma-Aldrich) under microwave heating. Endogenous peroxidase activity was quenched by incubation with methanolic hydrogen peroxide for 30 minutes. After permeabilized with 0.5% Triton X-100 (Sigma-Aldrich), the slides were blocked with normal donkey serum (Jackson ImmunoResearch) diluted in PBS (1:10) for 1 hour, after which they were reacted with anti-cleaved caspase-3 (1:250, Cell signaling, 9661s, clone Asp175), anti-Ki-67 (1:500, Abcam, ab16667, clone SP6), anti-Mucin 2 (1:1000, Abcam, ab97386), anti-lysozyme (1:1000, Thermofisher, PA5-16668), anti-Olfm4 (1:1000, Cell signaling, 39141, clone D6Y5A), anti-HSP70 antibody (1:10000, Enzo, ADI-SPA-810, clone C92F3A-5), anti-IL17A (1:1000, Abcam, ab79056), and anti-TNFα antibody (1:250, R&D systems, AF-210-NA) overnight at 4°C. A Biotin-SP AffiniPure Donkey Anti-Rabbit IgG (H+L) Jackson ImmunoResearch, 711-065-152), or Biotin-SP AffiniPure Donkey Anti-Mouse IgG (H+L) (Jackson ImmunoResearch, 715-065-150), or Biotin-SP AffiniPure Donkey Anti-Goat IgG (H+L) (Jackson ImmunoResearch, 705-065-147) diluted in PBS (1:200) was then added to the slides and incubated for 2 hours. After washing with PBS, a VECTASTAIN ABC kit (Vector laboratories) was added to the slides. After 1 hour, the tissues were stained with a DAB substrate kit (Vector laboratories), and the nuclei were counterstained using Harris hematoxylin (YD Diagnostics. The images were acquired with a Pannoramic Scan II scanner (3DHISTECH Ltd).

Immunofluorescence staining

Paraffin-embedded mouse ileal tissues were sectioned at a thickness of 4 ㎛. After deparaffinization and rehydration, antigen retrieval was performed by heating the sections in a microwave in citric acid buffer (pH 6.0) or Tris-EDTA buffer (pH 9.0). The tissues were then incubated with methanolic hydrogen peroxide for 30 minutes and treated with 0.5% Triton X-100 (Sigma-Aldrich, T8787) in PBS for 15 minutes. The sections were blocked with normal donkey serum (Jackson ImmunoResearch, 017-000-121) diluted 1:10 in PBS for 1 hour at room temperature. They were then reacted with the following primary antibodies overnight at 4°C: anti-NFAT5 (1:200, Santa Cruz, sc-398171; or 1:500, Novus Biologicals, NB120-3446), anti-Mucin 2 (1:500, Abcam, ab97386), anti-lysozyme (1:500, ThermoFisher, PA5-16668), anti-Olfm4 (1:500, Cell Signaling, 39141, clone D6Y5A), anti-sucrase-isomaltase (1:200, NSJ Bioreagents, RQ4659), anti-ZO-1 (1:500, BiCell scientific, 00236, clone F11 or 1:500, Proteintech, 21773-1-AP), anti-Occludin (1:500, Bicell scientific, 00241n), and anti-E-cadherin antibody (1:1000, R&D Systems, AF748). After washing with PBS, appropriate Alexa Fluor-conjugated secondary antibodies (Alexa 488, Alexa 594, or Alexa 647; Invitrogen, A21202, A21206, A21207, A21209, A21447) were treated for 2 hours at room temperature. The nuclei were stained with 4′,6-diamidino-2-phenylindole (DAPI; Roche, 70508621), and the slides were mounted using Fluoromount-G (SouthernBiotech, 0100-01). The images were acquired using an LSM 900 confocal microscope (Carl Zeiss) and processed with ZEN lite software (Carl Zeiss).

In vivo EdU incorporation assay

Mice were sacrificed 4 and 48 hours after intraperitoneal (i.p.) injection of 1 mg/mouse 5-ethynyl-2'-deoxyuridine (EdU; Thermofisher, A10044). Colon tissues were collected, fixed in 4% (w/v) paraformaldehyde in PBS (Biosesang, P2184), paraffin-embedded, and sectioned. After deparaffinization and hydration, tissues were stained using Click-iT EdU Imaging Kits (Invitrogen, C10337) according to the manufacturer′s instructions. The nuclei were stained with 4',6-diamidine-2′-phenylindole dihydrochloride (DAPI; Roche, 70508621) and mounted with Fluoromount-G (SouthernBiotech, 0100-01). Images were observed and analyzed using a LSM 900 confocal microscope (Carl Zeiss) and ZEN lite (Carl Zeiss).

RNA extraction and quantitative real-time PCR

RNA was extracted from colon tissues and small/ large intestinal epithelial cells using TRIzol (Invitrogen, 15596-018) and RNeasy Mini Kit (QIAGEN, 74106) according to the manufacturer’s instructions. cDNA was synthesized using RevertAid Reverse Transcriptase (ThermoFisher, EP0441) and oligo (dT) primer (Invitrogen). qRT-PCR was performed using SYBR Green PCR mixture (BioRad, 1708880) on a CFX96 real-time PCR system. The mRNA expression levels of the target genes were normalized to *GAPDH*. The specific primer sequence is listed in **Supplemental Table 5**.

Isolation of intestinal epithelial cells

The ileum and colon were dissected and longitudinally opened, free of Peyer’s patches and mesenteric fat. The tissues were sliced into < 5 mm size and washed with sHBSS [HBSS supplemented with 2.5% FBS]. Next, the tissues were incubated again in sHBSS supplemented

with 1 mM DTT (DL-Dithiothreitol) (Sigma, 43819) for 15 minutes at RT. After washing with sHBSS, the tissues were gently rocked in PBS containing 10 mM EDTA (Biosesang, E2002) for 30 minutes at RT. Intestinal epithelial cells were subsequently isolated by vigorous shaking for 1 minute. The detached cells were passed through a 100 μm pore strainer and collected by centrifugation at 1500 rpm for 3 minutes at RT.

Microarray analysis

Microarray analysis was conducted using the Affymetrix Mouse Gene 2.0 ST Array according to the manufacturer's instructions (Macrogen Inc., Seoul, South Korea). Briefly, epithelial cells were isolated from the colon and ileum of three *Nfat5*$^{\text{IEC-KO}}$ and three *Nfat5*$^{\text{fl/fl}}$ mice, and total RNA was prepared using the RNeasy Mini Kit (Qiagen). RNA purity and integrity were assessed using the ND-1000 Spectrophotometer (NanoDrop) and Agilent 2100 Bioanalyzer (Agilent Technologies), respectively. After cDNA synthesis, sense cDNA was fragmented and labeled with biotin using the GeneChip Whole Transcriptome PLUS Reagent Kit (Affymetrix). The labeled DNA targets were hybridized to the Affymetrix GeneChip Mouse Gene 2.0 ST array (Affymetrix) and scanned using a GCS3000 Scanner (Affymetrix). The data were summarized and normalized using the robust multi-array average (RMA) method implemented in Affymetrix® Power Tools (APT). After scanning, the quality of the raw microarray data was verified. Subsequently, the data were normalized and analyzed using Transcriptome Analysis Console (TAC) software version 4.0.0 (Thermo Fisher Scientific) along with the mouse reference genome (MoGene-2_0-st-v1.na36.mm10.transcript.csv) to generate a list of differentially expressed genes (DEGs). DEGs were selected based on the following criteria: fold change < -1.5 and > 1.5, p-value < 0.05, and false discovery rate < 0.1.

Biological-term classification and enrichment analysis of gene clusters were performed using clusterProfiler R software (1). Volcano plots were generated using the R package EnhancedVolcano with a *p*-cutoff = 0.05 and FC cutoff = 0.58 (log2 scaled) (2). For the heatmap visualization of DEGs, samples were normalized to z-scores and plotted using the pheatmap package in R.

Single-cell RNA sequencing analysis based on public database

To evaluate 'NFAT5 signature' according to cell types in human intestinal tissue, we arbitrarily defined 524 downregulated DEGs in the small intestine cells of *Nfat5*$^{\text{IEC-KO}}$ mice compared to *Nfat5*$^{\text{fl/fl}}$ mice as the 'NFAT5 signature'. The single-cell RNA-sequencing dataset of human intestinal tissues was obtained from a public database (GSE116222) (3). The classification of cell types in intestinal epithelial cells (IECs) was conducted by filtering raw count data using the scANVI model, a semi-supervised variational autoencoder. This process involved reference mapping and label transfer after training on reference data, followed by latent representation and label prediction through UMAP-based graph embedding. The Z-scores of 'NFAT5 signature' in each cell type were calculated using the 'tl.score_genes' function from the Scanpy package. Density plots of cells with high *Nfat5* signature scores were drawn using the tl.embedding_density and pl.embedding_density functions.

Western blot

Total proteins were extracted from IECs or cultured cell lysates using a RIPA lysis buffer (Biosesang, RC2002-050-00) supplemented with 1 mmol/L PMSF (Sigma-Aldrich, P7626-1G) and 1 mol/L DTT (Promega, V3151). The protein lysates were quantified using a Pierce™ BCA Protein Assay Kit (Thermo Fisher Scientific, 23227) according to the

manufacturer's protocol. Total proteins (50 - 60 μg) were mixed with Laemmli's sample buffer (Elpis, EBA-1052) and incubated at 90°C for 5 minutes. Proteins were then separated on a 6 - 10% SDS-PAGE gel and transferred onto an activated PVDF membrane (Millipore, IPVH00010). After incubation for 1 hour in blocking solution (5% (*w/v*) skim milk in TBS-0.1% (v/v) tween-20), the membrane was incubated with the primary antibody, including anti-ZO-1 (1:200, Thermo Fisher Scientific, 61-7300), anti-NFAT5 (1:1000, Abcam, ab3446), anti-HSP70 (1:3000, Enzo, ADI-SPA-810, clone C92F3A-5), anti-β-tubulin (1:1000, Abcam, ab15568), anti-GAPDH antibody (1:1000, Santa Cruz, sc-32233, clone 6C5), and anti-β-actin (1:1000, Santa Cruz, sc-47778, clone C4), overnight at 4°C. The membrane was washed and incubated again with anti-rabbit IgG-HRP (1:1000, Santa Cruz, sc-2357) or anti-mouse IgG-HRP (1:5000, Thermo Fisher Scientific, S31430) for 2 hours. After washing with TBS-0.1% Tween-20, the signals on the membranes were visualized using a chemiluminescence detection system (Promega). The expression of target proteins was normalized relative to that of β-actin using an ImageJ.

DNA extraction and next-generation sequencing of 16S rRNA

Feces and cecum contents were collected from 10-week-old male mice separately housed for more than 6 weeks. Total bacterial DNA was extracted using an E.Z.N.A.® Stool DNA Kit (Omega Bio-Tek, D4015-02) according to the manufacturer's instructions with an additional extensive bead-beating lysis step. The 16S ribosomal RNA amplicons (V3-V4 region) were prepared following the Illumina 16S metagenomic sequencing library preparation protocol. The V3-V4 regions of the bacterial 16S rRNA were amplified using 341-F (CCTACGGGNGGCWGCAG) / 805-R (GACTACHVGGGTATCTAATCC) primers (Cosmogenetech) and OneTaq Hot Start 2X Master Mix (New England Biolabs, M0484L).

The amplicons were quantified using a GenNext NGS Library Quantification Kit (Toyobo, NLQ-101) according to the manufacturer's instructions, and sequencing was performed on an Illumina Miseq system with a 250-base paired-end platform using a MiSeq Reagent Kit v2 (500-cycles) (Illumina, MS-102-2003).

Bioinformatics analysis

The generated raw data underwent preprocessing steps, including adapter removal and quality trimming, utilizing Cutadapt ver. 2.10 with default parameters (4). Cleaned paired-end reads were subsequently merged using PEAR ver. 0.9.6 (5), and the merged sequences were imported into the QIIME 2 platform ver. 2020.8 for denoising through the application of the DADA2 package (6, 7). Following denoising, the calculation of alpha-diversity metrics, such as Chao1, utilizing singletons, was deemed inappropriate. Thus, the Observed features index was used as an alpha-diversity, estimating microbial richness based on the number of unique bacterial taxa observed in a sample. Additionally, the Shannon index, which captures both the richness and evenness of the bacterial taxa present, was quantified as another alpha diversity. Non-metric multidimensional scaling (NMDS) plots, which intuitively show major bacterial compositional differences between each sample, were generated and permutational analysis of variance (PERMANOVA) was conducted using PRIMER 7 software ver. 7.023 (PRIMER-e, New Zealand) (8). Taxonomic assignment against the SILVA database ver. 132 was performed using the integrated analysis tools within QIIME 2 (9). Moreover, we identified the key bacterial genera that contributed significantly to distinguishing *Nfat5*$^{+/-}$ mouse feces from WT feces, using Linear discriminant analysis Effect Size (LEfSe) on Galaxy server 2.0 (http://galaxy.biobakery.org/) (10).

Crypt isolation and small Intestinal organoid cultures

The duodenum of the mouse small intestine was removed, cut open longitudinally, and flushed with PBS. The villi part of the intestine was gently scraped with coverslip glass and cut into small pieces (0.5 cm). The intestinal tissues were washed with PBS using a 10 mL serological pipette more than eight times until the supernatant became clear. The tissues were incubated in PBS containing 2.5 mM EDTA (Biosesang) for 30 minutes at 4°C, after which the solution was removed and the tissues were resuspended in 10 mL of PBS containing 10% FBS. Intestinal crypts were collected 4 times by vigorously shaking and passing through a 70-μm cell strainer (Falcon, 352350). The resultant crypt cells (in fraction 3 or 4) that had passed through were spun down at 300 g for 5 minutes at 4°C, washed twice with DMEM/F12 (Welgene, LM002-04) containing 2% penicillin-streptomycin (100 U/mL, Gibco, 15070063), and centrifuged again at 100 g for 5 minutes at 4°C. A total of 200 to 400 crypts per well were resuspended in Matrigel (Corning, 356231) and then cultured in Intesticult Organoid Growth Medium (Stemcell Technologies, 6005), supplemented with 2% penicillin-streptomycin. The cells were seeded and cultured in at least five wells per mouse group. After a 5-day culture period, the number of organoids was counted. Random non-overlapping images of the organoids were captured under a Leica DMI6000 B microscope. The organoid surface area was measured using Leica LAS X software.

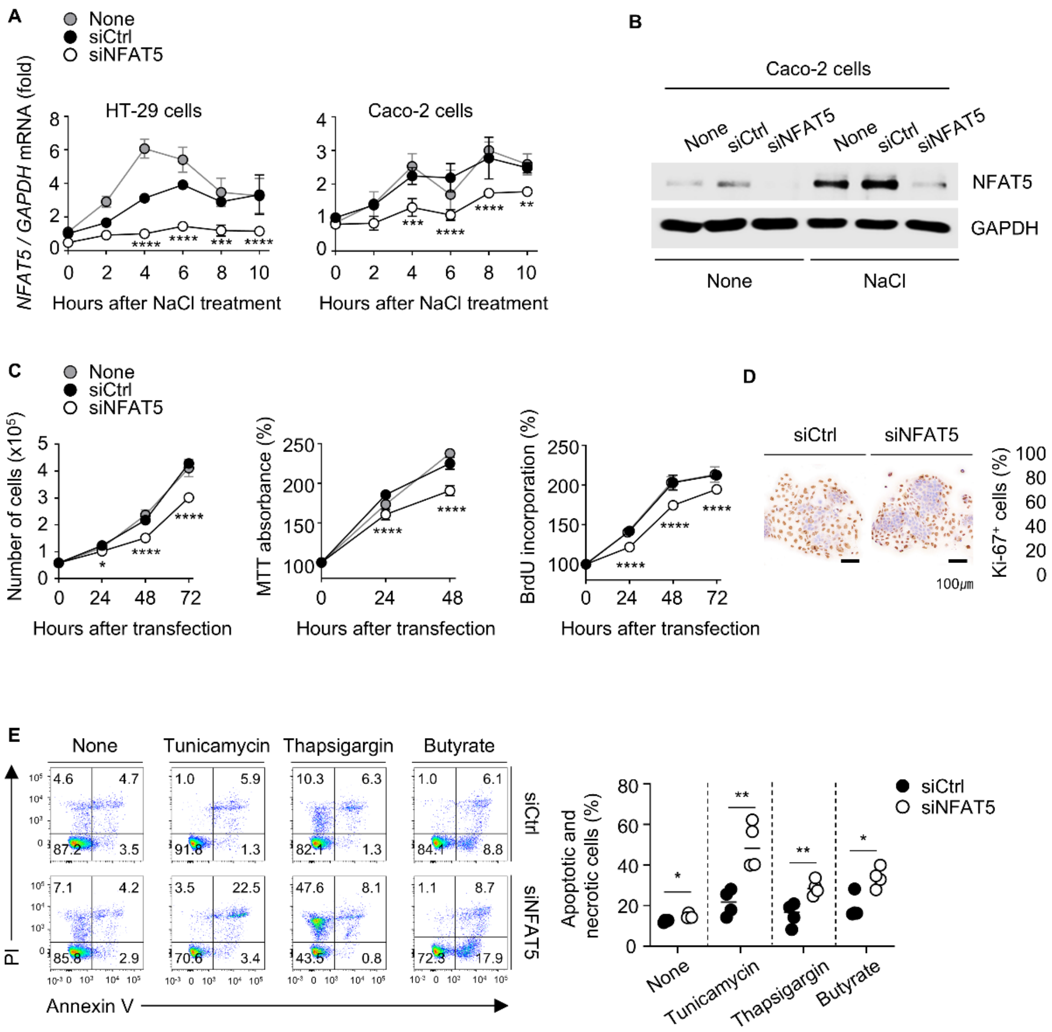

Supplemental Figure 1. Effect of NFAT5 on the proliferation and survival of IECs.

(**A** and **B**) Relative mRNA (**A**) and protein (**B**) expression levels of NFAT5 in HT-29 (**A**, left) and Caco-2 (**A**, right, and **B**) cells treated with 100 mM NaCl (hyperosmotic stimuli) for the indicated times (**A**) or 24 hours (**B**) after transfection with control siRNA (siCtrl) or *NFAT5* siRNA (siNFAT5), respectively, for 48 hours. *Gapdh* mRNA and GAPDH protein were used as internal controls. (**C** and **D**) Proliferation of Caco-2 cells transfected with siCtrl or siNFAT5 was assessed at the indicated time points using trypan blue exclusion (left), MTT (middle), and BrdU incorporation assays (right) (**C**). Representative images of Ki-67 immunocytochemistry

and corresponding quantified data are presented (**D**). (**E**) Role of NFAT5 in the apoptosis of Caco-2 cells. Caco-2 cells were either untreated or treated with tunicamycin, thapsigargin, or butyrate for 24 hours after transfection with siCtrl or siNFAT5 for 48 hours. The cells were then stained with Annexin V and PI and analyzed using flow cytometry. The left panel displays representative plots, while the right panel shows the total frequency of apoptotic and necrotic cells (Annexin V^{-}/PI^{-}: live cells, Annexin V^{-}/PI^{+}: necrotic cells, Annexin V^{+}/PI^{-}: early apoptotic cells, and AnnexinV^{+}/PI^{+}: late apoptotic cells). Data are presented as mean ± SD (**A** and **C**), and as a line indicating the mean (**D**). *$p < 0.05$, **$p < 0.01$, ***$p < 0.001$, and ****$p < 0.0001$ by the Two-way repeated measures ANOVA test with Sidak's multiple comparisons test (**A**, **C**, and **E**) (between cells transfected with siCtrl and siNFAT5) and by the Mann-Whitney *U* test (**D**). Data shown in **A**-**E** are at least three independent experiments.

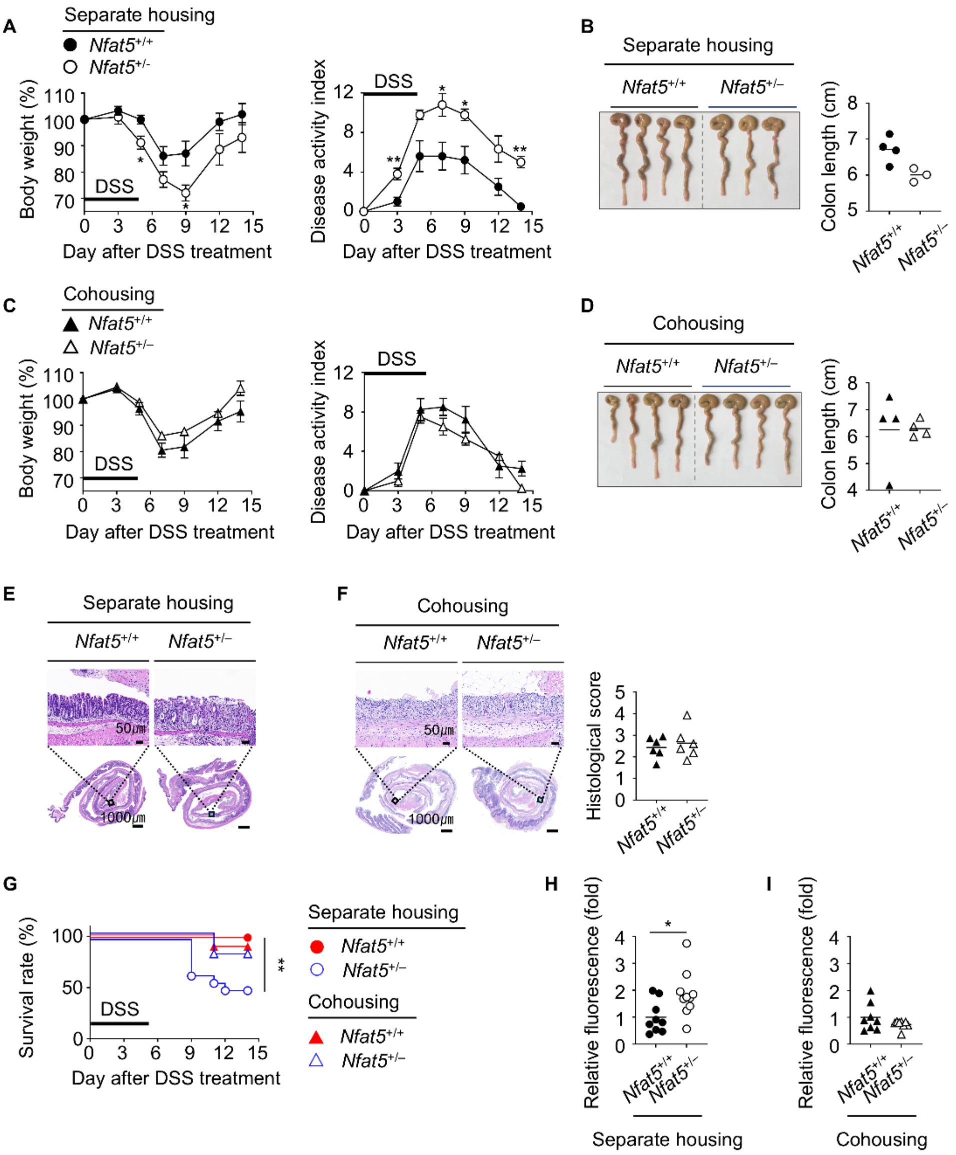

Supplemental Figure 2. Differential impact of separate housing versus cohousing on the severity of DSS-induced colitis in *Nfat5*$^{+/+}$ *and Nfat5*$^{+/–}$ mice.

Mice were provided *ad libitum* with DSS-containing water for 5 days, followed by fresh drinking water for the remainder of the experiment. (**A** and **C**) Body weight changes and disease activity index (DAI) scores of separately housed (**A**) or cohoused (**C**) *Nfat5*$^{+/+}$ and *Nfat5*$^{+/–}$ mice. (**B** and **D**) Macroscopic images and the length of colons from separately housed (**B**) or cohoused (**D**) *Nfat5*$^{+/+}$ and *Nfat5*$^{+/–}$ mice euthanized on day 14 following DSS treatment. (**E** and **F**) Representative H&E-stained images of distal colon tissues from separately housed (**E**) or cohoused (**F**) *Nfat5*$^{+/+}$ and *Nfat5*$^{+/–}$ mice collected on day 5 following DSS treatment.

Histological scoring was performed according to **Supplemental Table 3** (**F**). (**G**) The survival graph, derived from the pooled data of five independent experiments on DSS-induced colitis experiments comparing *Nfat5*$^{+/+}$ and *Nfat5*$^{+/-}$ mice that were either housed separately or cohoused. (**H** and **I**) Gut permeability assay of *Nfat5*$^{+/+}$ and *Nfat5*$^{+/-}$ mice, which were housed separately (**H**) or cohoused (**I**). Mice were provided with DSS water for 3 days and then fasted for 12 hours. Fluorescein isothiocyanate-dextran (FITC)-dextran (average molecular weight≈ 4 kDa) was administered to the mice via oral gavage. Four hours later, fluorescence signals were measured in the sera of the mice. Data are presented as mean ± SEM (**A** and **C**). Each dot represents an individual mouse, and the means are displayed as lines (**B**, **D**, **F**, **H**, and **I**). $*p < 0.05$, $**p < 0.01$, and $***p < 0.001$ by the Two-way repeated measures ANOVA test with Sidak's multiple comparisons test (**A** and **C**), the Mann-Whitney *U* test (**B, D**, **F**, **H,** and **I**), and the Log-rank test (**G**). Data in **A-D** are representative of three independent experiments; data shown in **F**, **H** and **I** are representative of two independent experiments.

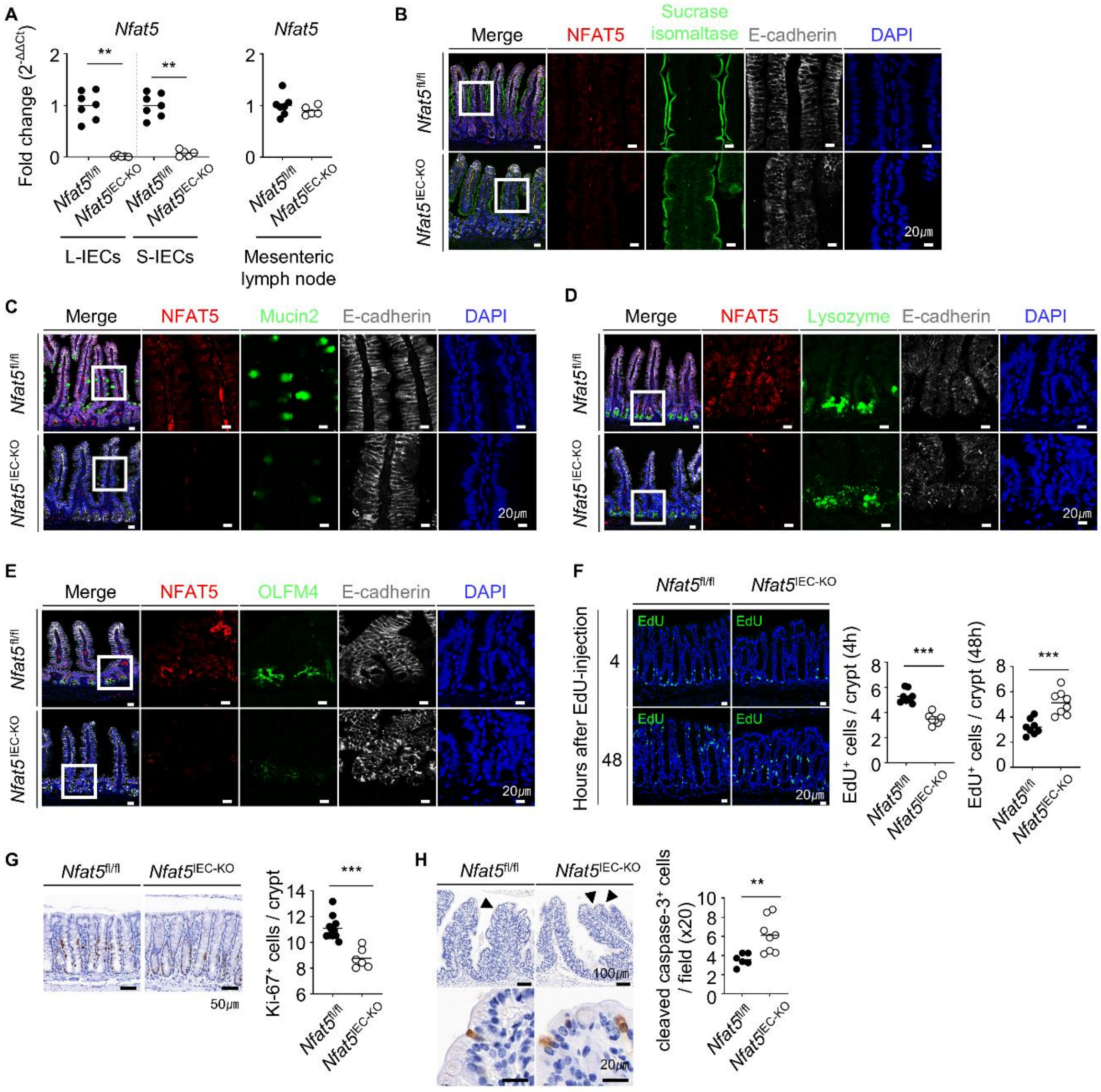

Supplemental Figure 3. Selective deletion of NFAT5 in IECs of *Nfat5*$^{\text{IEC-KO}}$ mice and its effects on epithelial cell proliferation and apoptosis.

(**A**) *Nfat5* mRNA expression was analyzed by qRT-PCR in large intestinal epithelial cells (L-IECs), small intestinal epithelial cells (S-IECs), and mesenteric lymph nodes of *Nfat5*$^{\text{fl/fl}}$ and *Nfat5*$^{\text{IEC-KO}}$ mice, normalized to *Gapdh* mRNA. (**B**–**E**) Colocalization of NFAT5 (red) with cell type-specific markers — Sucrase isomaltase for enterocytes (**B**), Mucin 2 for goblet cells (**C**), Lysozyme for Paneth cells (**D**), and OLFM4 for intestinal stem cells (**E**) (all in green) — was assessed in ileal tissues from *Nfat5*$^{\text{fl/fl}}$ and *Nfat5*$^{\text{IEC-KO}}$ mice by immunofluorescence staining. E-cadherin (white) was used as a marker for intestinal epithelial cells. Nuclei were

counterstained with DAPI (blue). Enlarged views of the boxed regions are provided to highlight areas of colocalization. (**F** and **G**) Cell proliferation in distal colonic tissues of *Nfat5*$^{fl/fl}$ and *Nfat5*$^{IEC\text{-}KO}$ mice was assessed by EdU incorporation assay (**F**) and Ki-67 immunohistochemistry (**G**). EdU^{+} cells were identified following intraperitoneal injection of EdU (1 mg) at specified time points. Representative images and quantified data for EdU and Ki-67 are presented. (**H**) Apoptotic cells were quantified in colon tissues of *Nfat5*$^{fl/fl}$ and *Nfat5*$^{IEC\text{-}KO}$ mice 3 days after initial DSS treatment, using immunohistochemical staining for cleaved caspase-3. Each dot represents an individual mouse, with means displayed as a line (**A**, and **F**-**H**). $^{**}p < 0.01$ and $^{***}p < 0.001$ by the Mann-Whitney *U* test (**A**, and **F**-**H**). Data in **A**-**H** are representative of two independent experiments.

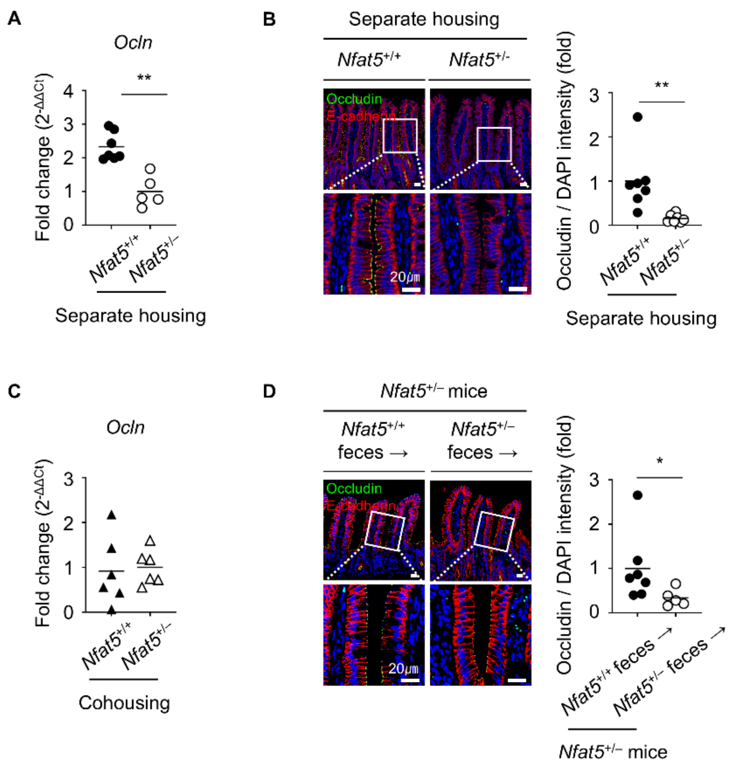

Supplemental Figure 4. Expression of Occludin (Ocln), a tight junction-associated protein, is reduced by *Nfat5* deficiency and exposure to feces of *Nfat5*$^{+/–}$ mice.

(**A** and **C**) Relative mRNA expression levels of *Ocln* in small intestinal epithelial cells (S-IECs) from separately housed (**A**) or cohoused (**C**) *Nfat5*$^{+/+}$ and *Nfat5*$^{+/-}$ mice, as quantified by qRT-PCR. *Gapdh* mRNA served as an internal control for normalization. (**B**) Immunofluorescence analysis of Occludin expression in ileal tissues from separately housed *Nfat5*$^{+/+}$ and *Nfat5*$^{+/-}$ mice, with representative images and quantitative data. Magnified views of the indicated boxed areas are presented in the lower panels. (**D**) Immunofluorescence analysis of ileal tissues from *Nfat5*$^{+/-}$ recipient mice that received fecal microbiota transplantation (FMT) from either *Nfat5*$^{+/+}$ or *Nfat5*$^{+/-}$ donor mice, stained for Occludin (green) and E-cadherin (red). Prior to FMT, recipient mice were administered an antibiotic cocktail for 3 weeks. Subsequently, feces from *Nfat5*$^{+/+}$ or *Nfat5*$^{+/-}$ mice were transplanted five times at two-day intervals. Nuclei were counterstained with DAPI (blue). Representative merged images and the corresponding graphs are shown. Each dot represents an individual mouse, and mean values are displayed as a line. **p* < 0.05 and ***p* < 0.01 by the Mann-Whitney *U* test. Data shown in **A-D** are representative of two independent experiments.

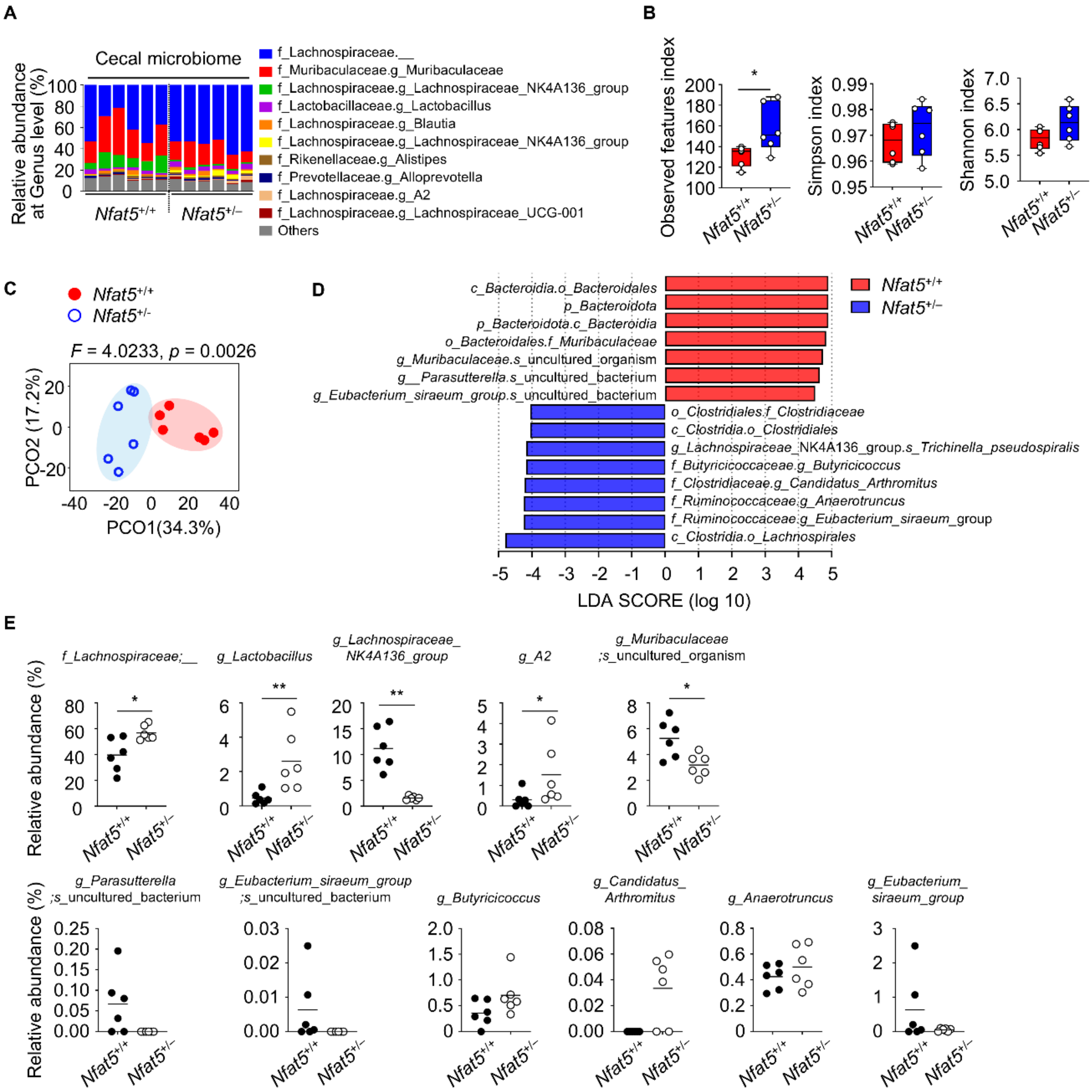

Supplemental Figure 5. Changes in the composition of cecal microbiota by NFAT5 deficiency.

Cecal microbiome in separately housed *Nfat5*$^{+/+}$ and *Nfat5*$^{+/-}$ mice (n = 6 per group) was analyzed using 16S rRNA amplicon sequencing. (**A**) Relative abundance of cecal bacteria at the genus level. (**B**) α-diversity: Observed features, Simpson's, and Shannon's indices. Data are presented as box-and-whisker plots, displaying the minimum and maximum values, with a line indicating the median. (**C**) β-diversity: PCoA plot at the ASV level. Pseudo-*F* and *p*-values were analyzed using PERMANOVA. (**D**) LEfSe analysis: Bacterial taxa with an LDA score greater than 4 represent microbial species with significant differences between groups. (**E**)

Relative abundance of each taxon suggested by the results of (**A**) and (**D**). Each dot represents an individual mouse, and means are displayed as lines. *$p < 0.05$ and **$p < 0.01$ by the Mann-Whitney *U* test.

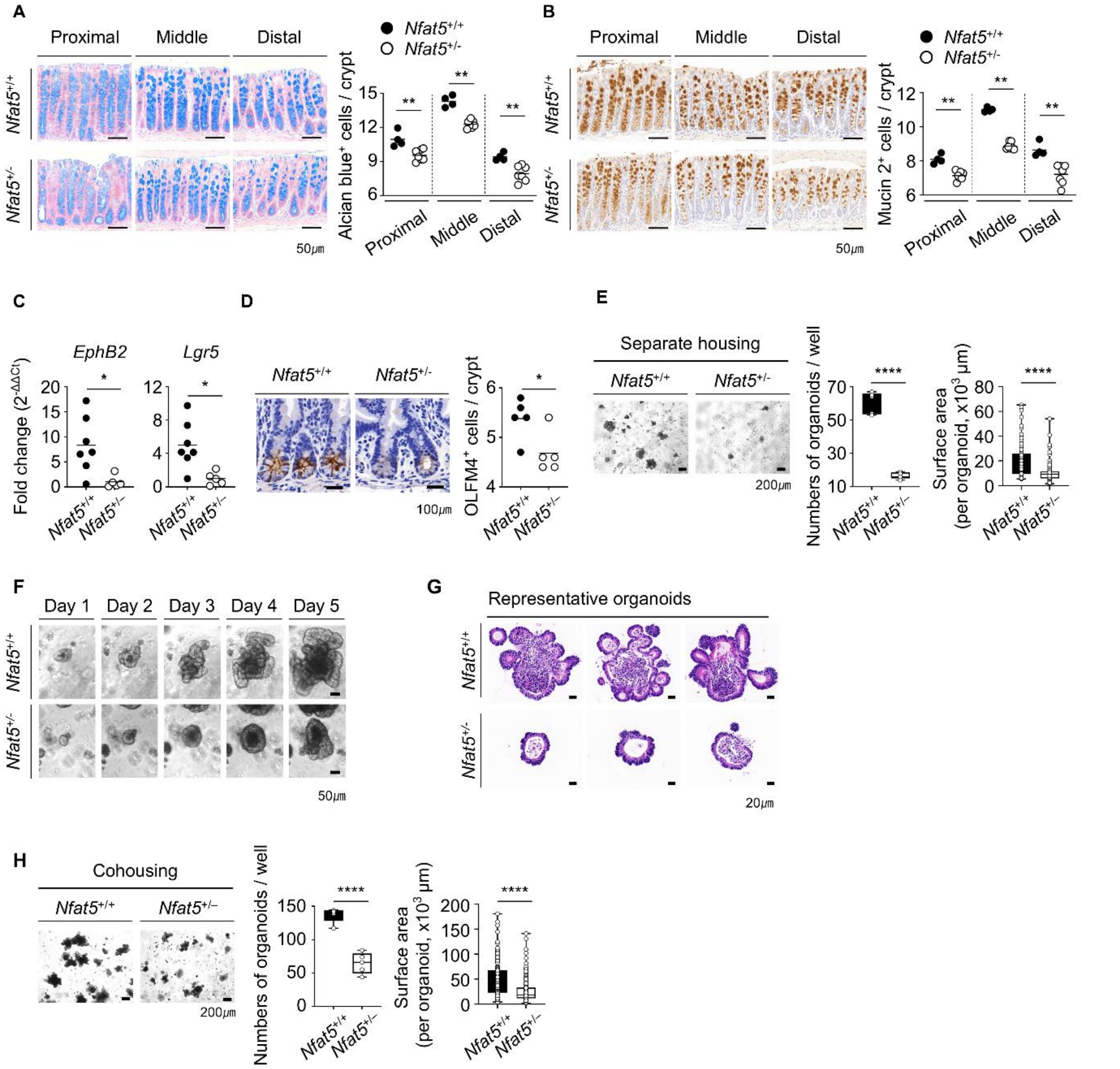

Supplemental Figure 6. NFAT5 affects the production of mucin from the goblet cells through the regulation of epithelial regenerative capacity.

(**A** and **B**) Mucin-producing cells in the colon tissues of separately housed *Nfat5*$^{+/+}$ and *Nfat5*$^{+/-}$ mice, as assessed by Alcian blue staining (**A**) and Mucin 2 immunostaining (**B**). (**C**) Relative mRNA expression levels of *EphB2* and *Lgr5* in small intestinal epithelial cells from separately housed *Nfat5*$^{+/+}$ and *Nfat5*$^{+/-}$ mice. *Gapdh* mRNA was used as an internal control for normalization. (**D**) Immunohistochemistry of OLFM4 in ileal tissues of separately housed *Nfat5*$^{+/+}$ and *Nfat5*$^{+/-}$ mice. Representative images are shown in the left panel, with corresponding quantitative data presented in the right panel. (**E-H**) Organoids derived from

small intestinal crypts of separately housed (**E**-**G**) or cohoused (**H**) *Nfat5*$^{+/+}$ and *Nfat5*$^{+/-}$ mice were generated and cultured in Matrigel for 5 days. Crypts isolated from each mouse were seeded into five wells, and the number and size of the organoids formed in these wells were quantified (**E** and **H**); multiple images were captured from each well, and the surface area of all organoids within the images was also measured. Representative images of organoid culture wells (left), along with the corresponding quantitative graphs (right), are presented (**E** and **H**). Additionally, representative time-lapse images (**F**) and H&E-stained sections (**G**) of organoids are shown. Each dot represents an individual mouse, with group means indicated by horizontal lines (**A-D**). Data are presented as box-and-whiskers plots (minimum-to-maximum, line at median) (**E** and **H**). *$p < 0.05$, **$p < 0.01$, and ****$p < 0.0001$ by the Mann-Whitney *U* test (**A-D**) and unpaired *t*-test (**E** and **H**). Data shown in **A**-**D** are representative of two independent experiments; data in **E** and **H** are at least three independent experiments

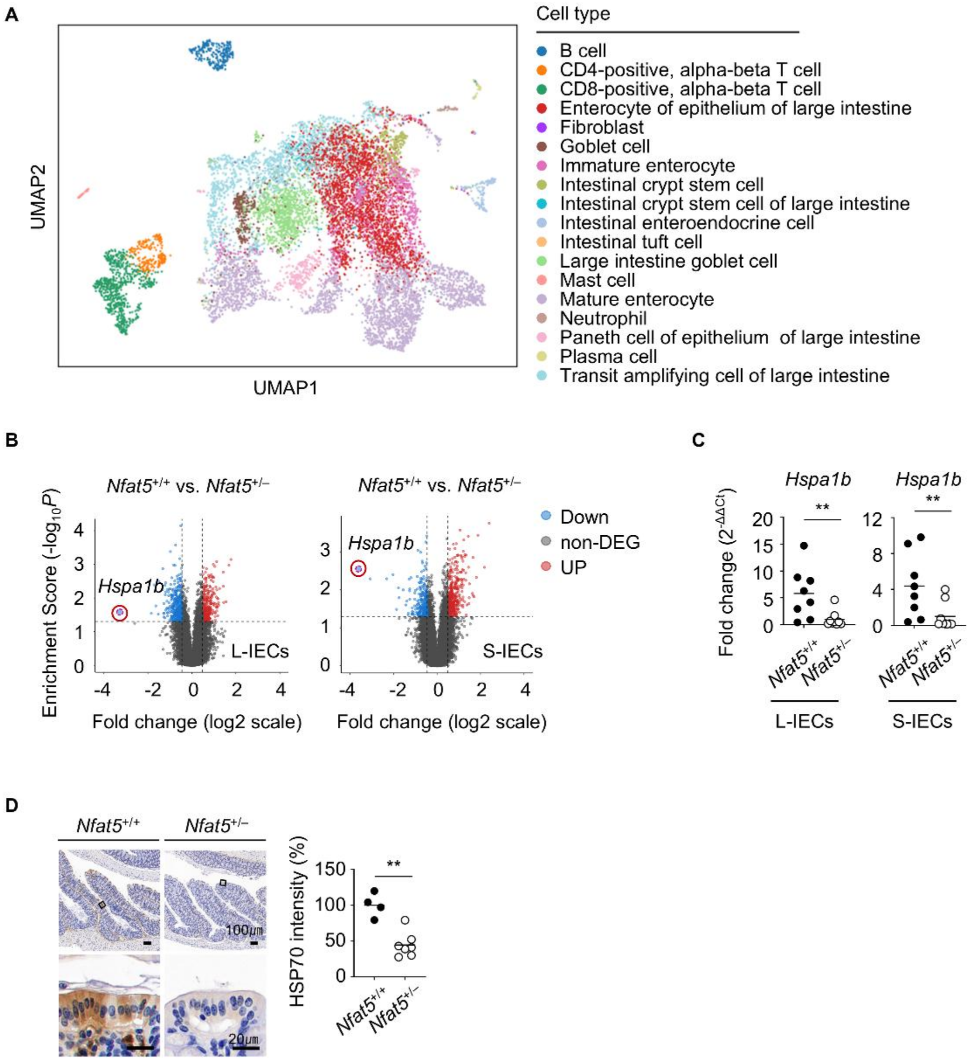

Supplemental Figure 7. Transcriptomic profiling of human and mouse IECs reveals HSP70 as a major NFAT5-regulated gene.

(**A**) UMAP plot of single-cell RNA-seq data from human colonic epithelium showing 18 clusters. The scRNA-seq data were obtained from a published database (GEO No: GSE116222). (**B**) Transcriptome analysis was performed using microarray on large intestinal epithelial cells (L-IECs) and small intestinal epithelial cells (S-IECs), isolated from *Nfat5*$^{+/+}$ and *Nfat5*$^{+/–}$ mice (n = 3 per group). Volcano plots illustrate the fold change and significance levels of differentially expressed genes (DEGs). Red represents genes that are upregulated in *Nfat5*$^{+/–}$ mice compared with *Nfat5*$^{+/+}$ mice, along with their Z-scores, whereas blue indicates

downregulated genes and their corresponding Z-scores. (**C**) Relative expression levels of *Hspa1b* mRNA in large intestinal epithelial cells (L-IECs, left) and small intestinal epithelial cells (S-IECs, right) isolated from *Nfat5*$^{+/+}$ and *Nfat5*$^{+/-}$ mice. Expression levels were normalized to *Gapdh* mRNA. (**D**) HSP70-expressing cells in colon tissues collected from *Nfat5*$^{+/+}$ and *Nfat5*$^{+/-}$ mice were identified by immunohistochemical (IHC) staining for HSP70. Representative IHC images (left) and corresponding quantified data (right) are shown. The lower panel displays enlarged views of the boxed areas. Each dot represents an individual mouse, and the means are displayed as lines (**C** and **D**). $^{**}p < 0.01$ by the Mann-Whitney *U* test. Data in **C** and **D** are representative of two independent experiments.

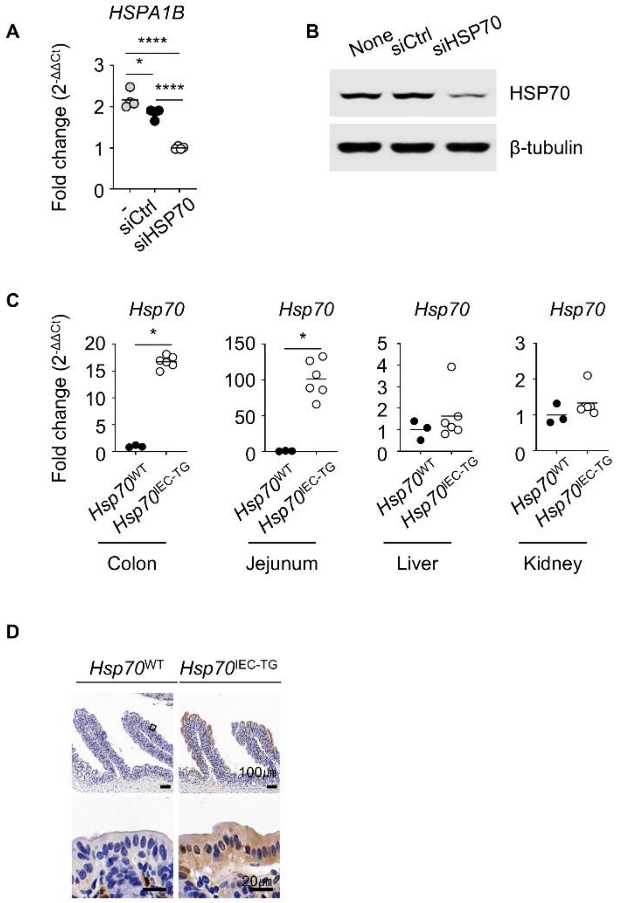

Supplemental Figure 8. HSP70 expression levels in *Hsp70*-knockdown cells and *Hsp70*$^{\text{IEC-TG}}$ mice.

(**A** and **B**) *HSPA1B* mRNA (**A**) and HSP70 protein (**B**) expression levels in HT-29 cells transfected with control siRNA (siCtrl) or *HSP70* siRNA (siHSP70) for 48 hours. *GAPDH* mRNA and β-tubulin protein were used as internal controls, respectively. (**C**) Relative expression levels of *Hspa1b* mRNA in colon, jejunum, liver, and kidney tissues isolated from *Hsp70*^{WT} and *Hsp70*$^{\text{IEC-TG}}$ mice, normalized to *Gapdh* mRNA. (**D**) Representative immunohistochemical images of HSP70 expression in colonic tissues of *Hsp70*^{WT} and *Hsp70*$^{\text{IEC-TG}}$ mice. Enlarged views of the boxed regions are shown in the lower panel. The mean values indicated by lines (**A**). Each dot represents an individual mouse, and the means are displayed as lines (**C**). *$p < 0.05$ and ****$p < 0.0001$ by the unpaired *t*-test (**A**) and Mann-Whitney *U* test (**C**). Data shown in **A**-**D** are representative of at least two independent experiments.

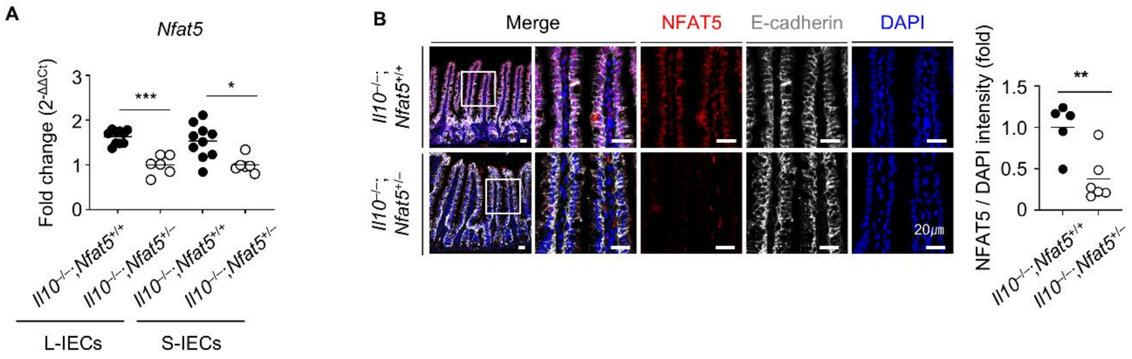

Supplemental Figure 9. NFAT5 expression in IECs of *Il10*$^{-/-}$; *Nfat5*$^{+/+}$ and *Il10*$^{-/-}$; *Nfat5*$^{+/-}$ mice.

(**A**) *Nfat5* mRNA expression levels in large intestinal epithelial cells (L-IECs) and small intestinal epithelial cells (S-IECs) collected from *Il10*$^{-/-}$; *Nfat5*$^{+/+}$ and *Il10*$^{-/-}$; *Nfat5*$^{+/-}$ mice, normalized to *Gapdh* mRNA expression levels. (**B**) NFAT5 protein expression in the epithelial layer of ileal tissues from *Il10*$^{-/-}$; *Nfat5*$^{+/+}$ and *Il10*$^{-/-}$; *Nfat5*$^{+/-}$ mice, as determined by immunostaining for NFAT5 (red) and E-cadherin (white), marking the intestinal epithelial cells. Nuclei were counterstained with DAPI (blue). Representative immunofluorescence images (left) and the corresponding graph (right) are shown. Each dot represents an individual mouse, and means are displayed as lines. *$p < 0.05$, **$p < 0.01$, and ***$p < 0.001$ by the Mann-Whitney *U* test. Data in **A** and **B** are representative of two independent experiments.

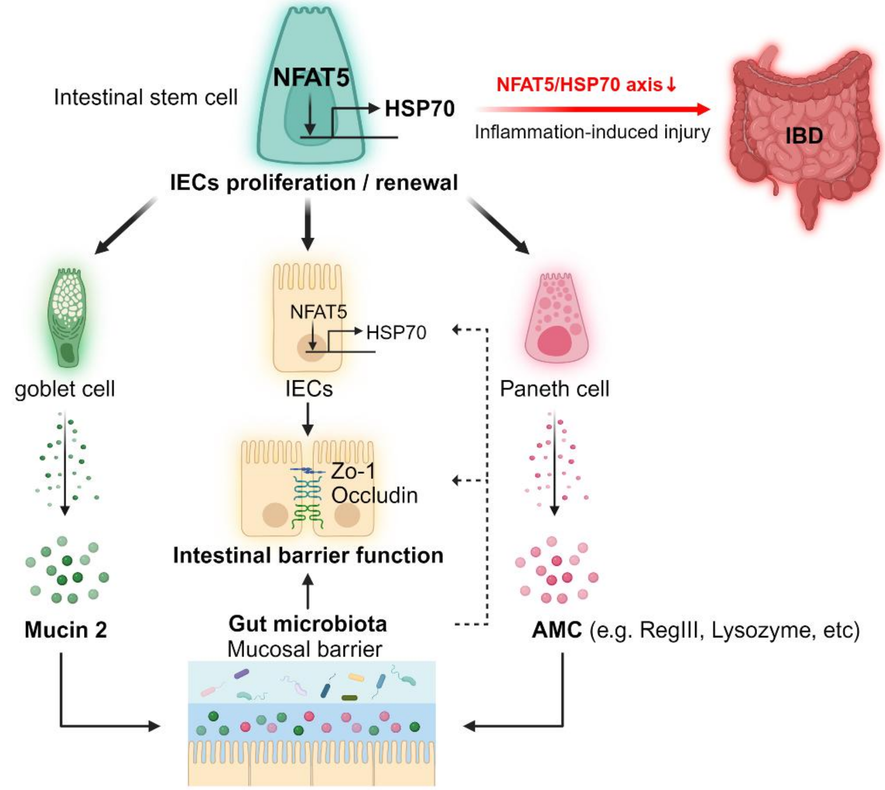

Graphical Abstract: Hypothetical model for the role of NFAT5 in maintaining gut homeostasis and protecting against colitis.

NFAT5 directly regulates the transcription of HSP70, which plays a crucial role in the proliferation and survival of IECs and the regenerative activity of ISCs. The NFAT5-HSP70 axis contributes to the production of Mucin 2 (Muc2) and AMCs by affecting the number of goblet and Paneth cells, respectively, as well as the renewal/survival of stem cells, thereby determining gut microbial composition. The gut microbiome, in turn, increases the expression of NFAT5 and HSP70, creating a positive feedback loop. The gut microbiome also influences tight junctions between epithelial cells by stimulating the expression of tight junction molecules, such as ZO-1 and Occludin. The enhancement of the intestinal barrier function facilitates the maintenance of gut homeostasis, resulting in protection against colitis development. In contrast, diminution and/or dysfunction of the NFAT5-HSP70 axis leads to a decrease in intestinal epithelial regenerative activity necessary for the generation of goblet and Paneth cells, resulting in dysbiosis of the gut microbiome. Dysregulation of the NFAT5-HSP70-gut microbiome axis then leads to a leaky gut, increasing susceptibility to colitis. HSP70: Heat-shock protein 70; ISC: Intestinal stem cell; IEC: Intestinal epithelial cell; ZO-1: Tight junction protein 1; AMC: Antimicrobial compound; IBD: Inflammatory bowel disease. This figure was created with BioRender.com.

Supplemental Table 1. Disease Activity Index scoring for the DSS-induced colitis model.

Score	Body weight loss (%)	Occult bleeding	Stool consistency
0	≤0	Normal	Normal
1	0-5	-	-
2	5-10	Slight bleeding	Loose stool
3	10-15	-	-
4	15-20	Gross bleeding	Diarrhea
5	>20	-	-

Supplemental Table 2. Disease Activity Index scoring for the *IL-10*$^{-/-}$ spontaneous colitis model.

Score	Stool consistency	Occult bleeding	Rectal prolapse
0	Normal	No blood	No prolapse
1	-	-	-
2	Loose stools	Positive occult blood	Prolapse evident only during defecation
3	-	-	-
4	Liquid stools	Gross bleeding	Prolapse evident all times

Supplemental Table 3. Histopathological scoring for the DSS-induced colitis model.

Score	Inflammation	Intestinal architecture
0	None	None
1	Mild	Focal erosion
2	Moderate	Focal ulcerations
3	Marked	Extended ulcerations

Supplemental Table 4. Histopathological scoring for the *IL-10*$^{-/-}$ spontaneous colitis model.

	Inflammation		Epithelial change
Score	Severity	Extent	
0	None	None	None
1	Minimal	Mucosa	Minimal hyperplasia
2	Mild	Mucosa, discontinuous extension into the submucosa	Mild hyperplasia with erosions
3	Moderate	Mucosa and submucosa	Moderate hyperplasia
4	Marked	Mucosa and submucosa	Marked hyperplasia
5	Marked	Transmural	Marked hyperplasia with multiple crypt abscesses

Supplemental Table 5. Primer sequences for real-time PCR

Host: Gene	Forward/ Reverse	Sequence (5'-3')	Reference
Mouse: *Il1b*	F	GTGGCTGTGGAGAAGCTGTG	This study
	R	GAAGGTCCACGGGAAAGACAC	
Mouse: *Il6*	F	TTCCATCCAGTTGCCTTCTTG	This study
	R	AGGTCTGTTGGGAGTGGTATC	
Mouse: *Il17a*	F	TCCAGAAGGCCCTCAGACTA	PMID: 30455690
	R	TTCATTGCGGTGGAGAGTC	
Mouse: *Tnfa*	F	TGAAGGGAATGGGTGTTCAT	This study
	R	TTGGACCCTGAGCCATAATC	
Mouse: *Zo-1*	F	CCACCTCTGTCCAGCTCT TC	This study
	R	ACACCGGAGTGATGGTTTTCTG	
Mouse: *Occludin*	F	ATGGCCTACTCCTCCAATGGC	This study
	R	CCCCACCTGTCGTGTAGTCT	
Mouse: *Mucin 2*	F	CATTTCTTGGGGCAGAGTGAG	PMID: 12080087
	R	GAATGTGAGAGGCTGCTGACC	
Mouse: *Reg3b*	F	CTCTCCTGCCTGATGCTCTT	This study
	R	GTAGGAGCCATAAGCCTGGG	
Mouse: *Reg3g*	F	TCAGGTGCAAGGTGAAGTTG	This study
	R	GGCCACTGTTACCACTGCTT	
Mouse: *Defa5*	F	ACTGAGGAGCAGCCAGGGGA	This study
	R	ACGCGTTCTCTTCTTTTGCAGCC	
Mouse: *Lyz1*	F	GAGACCGAAGCACCGACTATG	This study
	R	CGGTTTTGACATTGTGTTCGC	
Mouse: *EphB2*	F	CCATTGAACAGGACTACAGACTAC C	This study
	R	CACCGTGTTAAAGCTGGTGTAG	
Mouse: *Lgr5*	F	GGGAGCGTTCACGGGCCTTC	This study
	R	GGTTGGCATCTAGGCGCAGGG	
Mouse: *Olfm4*	F	CAGCCACTTTCCAATTTCACTG	PMID: 29203393
	R	GCTGGACATACTCCTTCACCTTA	
Mouse: *Nfat5* for *Nfat5*$^{+/-}$ mouse	F	AACCAGTGGTGTTGCAGGTA	This study
	R	AGGGAGTTGTATTTCGCCCAG	
Mouse: *Nfat5* for *Nfat5*$^{fl/fl}$ mouse	F	AACATTGGACAGCCAAAAGG	PMID: 25601839
	R	GCAACACCACTGGTTCATTA	
Mouse: *Hsp70* for *Hsp70*$^{IEC\text{-}TG}$ mouse	F	ACTGCCCTGATCAAGCGC	PMID: 19299581
	R	CGGGTTGGTTGTCGGAGTAG	

Mouse: *Hspa1b*	F	AGGTGCAGGTGAGCTACAAG	PMID: 18608577
	R	ATGATCCGCAGCACGTTGAG	
Mouse: *Gapdh*	F	AACTTTGGCATTGTGGAAGG	This study
	R	GGATGCAGGGATGATGTTCT	
Human: *NFAT5*	F	CCAGAAGTCATTTGCCTGGT	This study
	R	GATTCCAAGCCCACTCTTCA	
Human: *HSPA1B*	F	TGTCTTTGAGGTGGACTGTT	PMID: 31616780
	R	AGCCAGCTAATTACCATCAG	
Human: *TNFA*	F	CTCTTCTGCCTGCTGCACTTTG	PMID: 32579975
	R	ATGGGCTACAGGCTTGTCACTC	
Human: *GAPDH*	F	AAGGTGAAGGTCGGAGTCAA	This study
	R	AATGAAGGGGTCATTGATGG	

Supplemental References

1. Yu G, et al. clusterProfiler: an R package for comparing biological themes among gene clusters. *OMICS*. 2012;16(5):284-287.

2. Blighe K, et al. EnhancedVolcano: Publication-Ready Volcano Plots with Enhanced Colouring and Labeling. https://github.com/kevinblighe/EnhancedVolcano. Updated October 28, 2021. Accessed June, 26, 2023.

3. Parikh K, et al. Colonic epithelial cell diversity in health and inflammatory bowel disease. *Nature*. 2019;567(7746):49-55.

4. Martin M. Cutadapt removes adapter sequences from high-throughput sequencing reads. *EMBnet J*. 2011;17(1):3.

5. Zhang J, et al. PEAR: a fast and accurate Illumina Paired-End reAd mergeR. *Bioinformatics*. 2014;30(5):614-620.

6. Callahan BJ, et al. DADA2: High-resolution sample inference from Illumina amplicon data. *Nat Methods*. 2016;13(7):581-583.

7. Bolyen E, et al. Reproducible, interactive, scalable and extensible microbiome data science using QIIME 2. *Nat Biotechnol.* 2019;37(8):852-7.

8. Clarke KR, Gorley RN. PRIMER v7: user manual/tutorial 3rd ed. Plymouth, United Kingdom: Primer-E Ltd; 2015

9. Quast C, et al. The SILVA ribosomal RNA gene database project: improved data processing and web-based tools. *Nucleic Acids Res*. 2013;41(Database issue):D590-D596.

10. Segata N, et al. Metagenomic biomarker discovery and explanation. *Genome Biol*. 2011;12(6):R60.
