# Supplementary material for: NFAT5 dictates crosstalk between intestinal epithelial regenerative capacity and microbiota in murine colitis models
Source: J Clin Invest. 2025 Jul 15;135(18):e183093. doi: 10.1172/JCI183093 (PMC12435833; doi:10.1172/JCI183093)
Supplement: Unedited blot and gel images [file jci-135-183093-s143.pdf]

Uncropped blot for Figure 4D

Anti-ZO-1

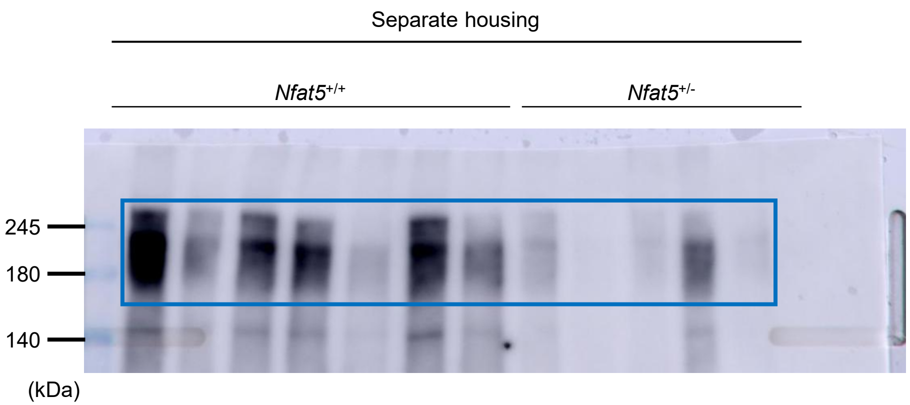

Anti-β-actin

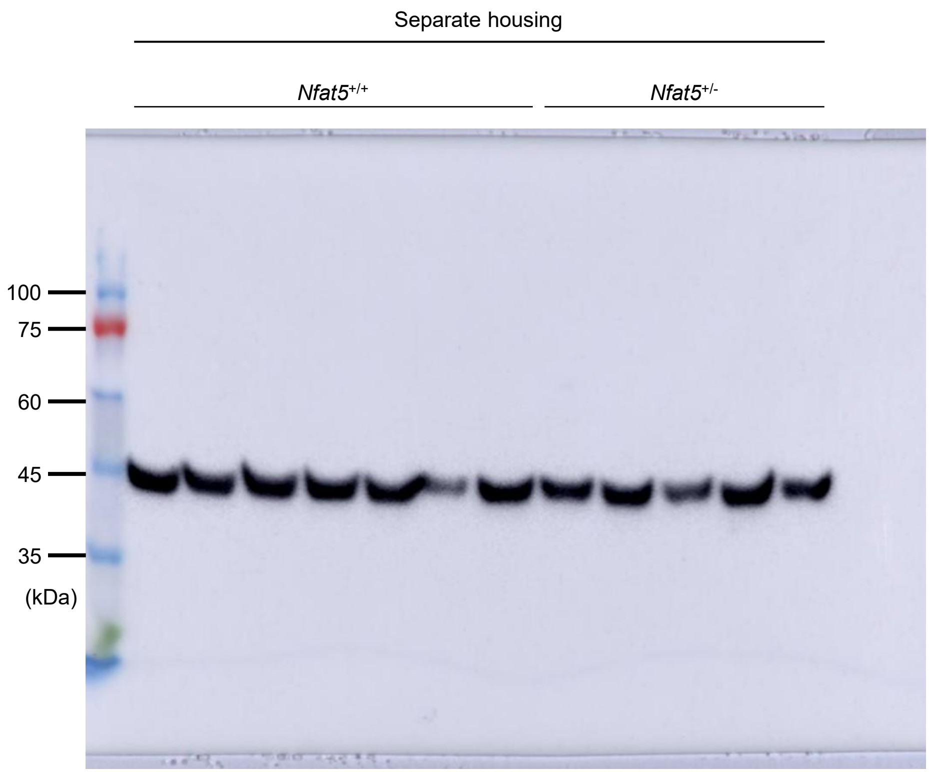

Uncropped image of the Western blot shown in Figure 4D

Uncropped blot for Figure 4G

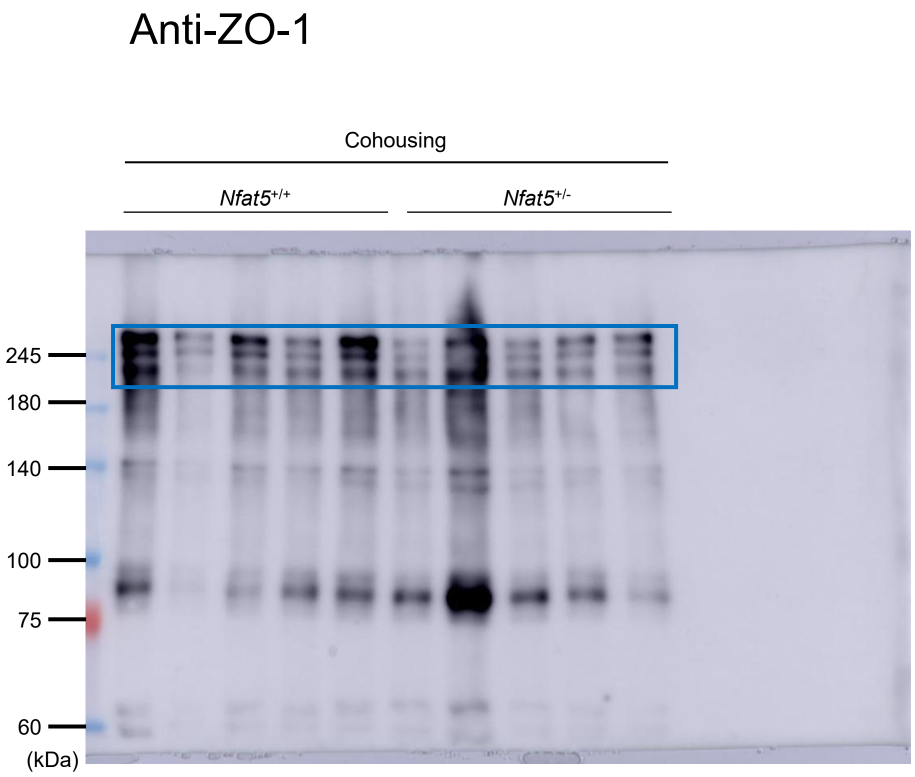

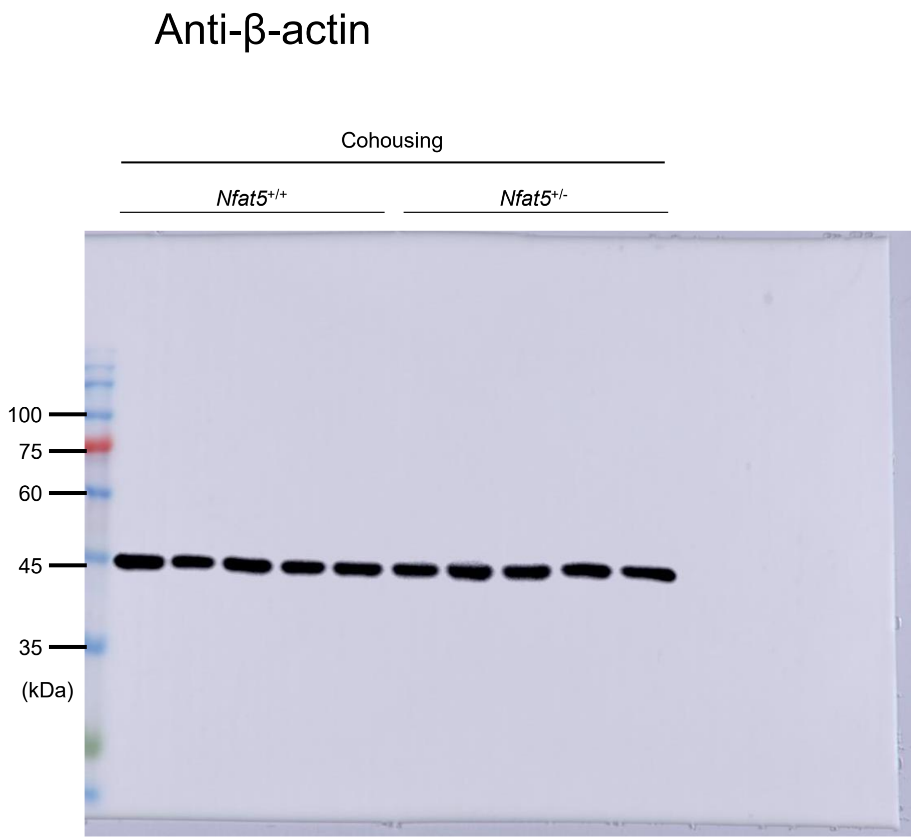

Uncropped image of the Western blot shown in Figure 4G

Uncropped blot for Figure 8B

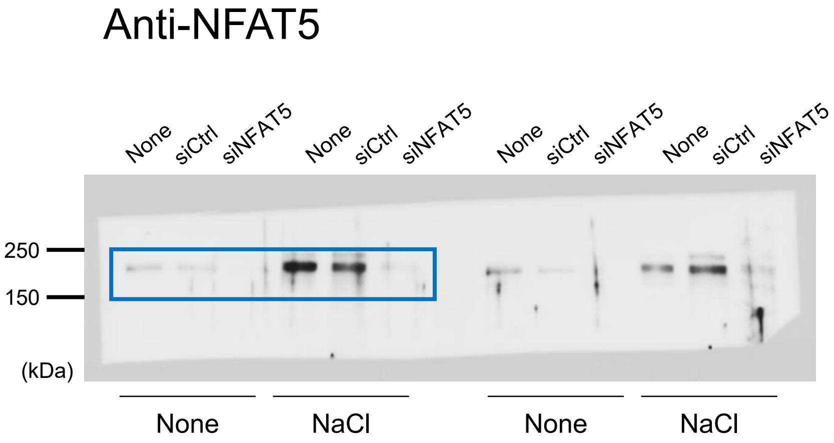

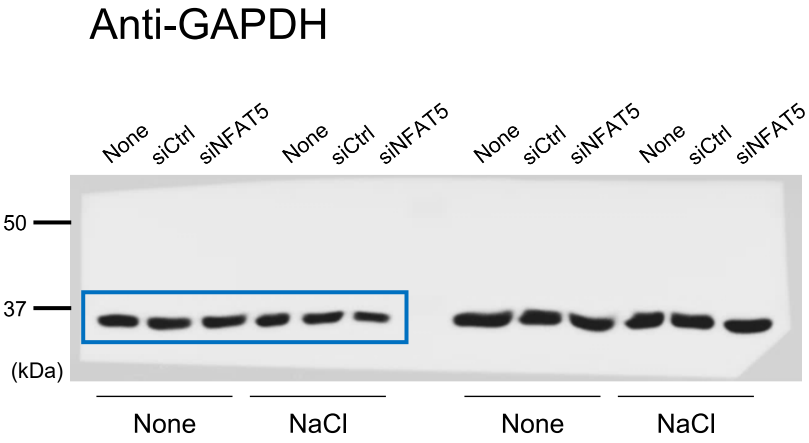

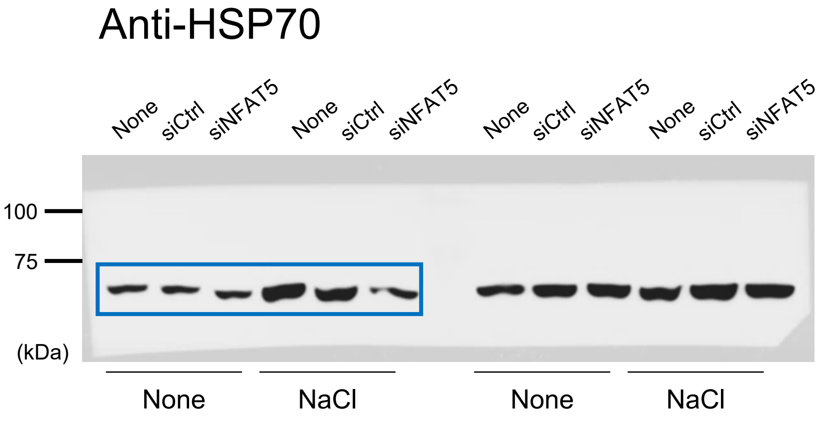

Uncropped image of the Western blot shown in Figure 8B

Uncropped blot for Supplemental Figure 1B

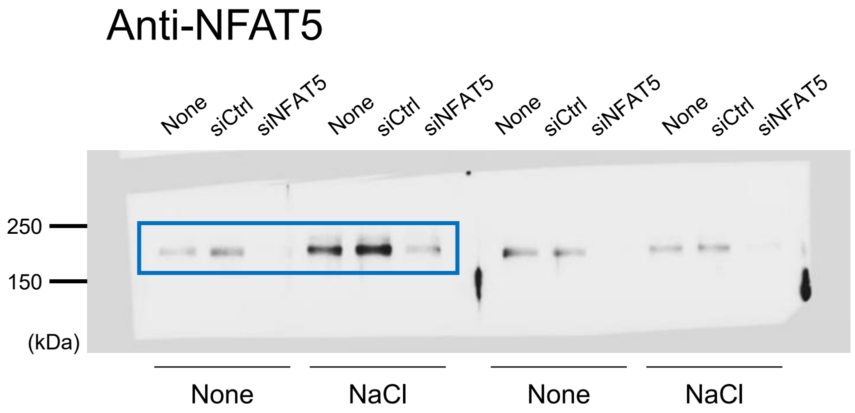

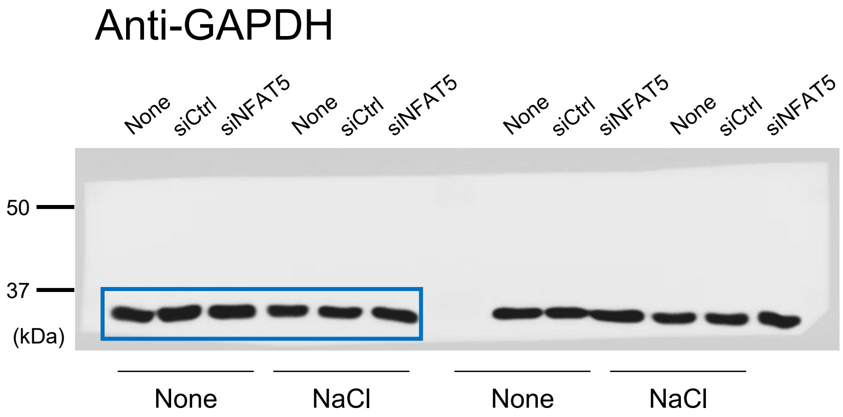

Uncropped image of the Western blot shown in Supplemental Figure 1B

Uncropped blot for Supplemental Figure 8B

Anti-HSP70

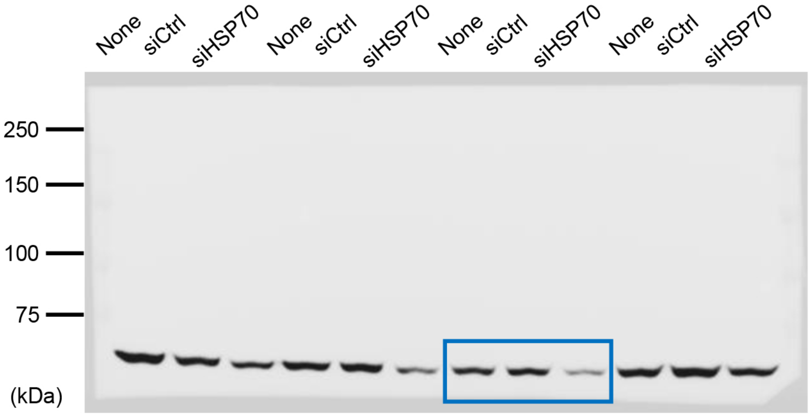

Anti-β-tubulin

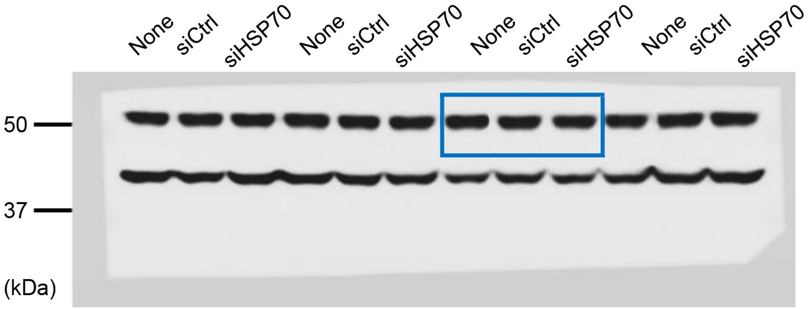

Uncropped image of the Western blot shown in Supplemental Figure 8B
